# Genome mining reveals the distribution of biosynthetic gene clusters in *Alternaria* and related fungal taxa within the family Pleosporaceae

**DOI:** 10.1186/s12864-025-11754-z

**Published:** 2025-07-21

**Authors:** Natalie E. Kim, Jeremy R. Dettman

**Affiliations:** 1 University of Guelph, 50 Stone Rd E, Guelph, ON N1G 2W1, Canada; 2 Agriculture and Agri-Food Canada, Ottawa Research and Development Centre, 960 Carling Avenue, Ottawa, ON K1A 0C6 Canada

**Keywords:** *Alternaria*, Biosynthetic gene cluster profile, Secondary metabolite, Genome mining, Comparative genomics

## Abstract

**Background:**

The advancement of whole genome sequencing techniques has led to the development of genome mining strategies that enable high-resolution research into fungal secondary metabolite (SM) biosynthesis. *Alternaria* species are producers of prominent SMs including virulence factors and mycotoxins that affect phytosanitation, food safety, and the economy. Here, we apply genome mining to identify a total of 6,323 biosynthetic gene clusters (BGCs) from 187 genomes: 123 *Alternaria* and 64 from seven other closely related genera in the family Pleosporaceae.

**Results:**

An average of 34 BGCs were detected per genome, with 29 on average for *Alternaria* genomes. The distribution of different BGC classes (e.g. polyketide synthases, non-ribosomal peptide synthetases) across taxa was investigated. BGCs were grouped into 548 gene cluster families (GCFs) revealing that while individuals within the same species may differ in their toxicological potential, the overall patterns of GCF presence/absence were also generally well correlated with phylogenomic patterns at higher taxonomic levels. Comparative genome analyses revealed that the divergent *Alternaria* sections *Infectoriae* and *Pseudoalternaria* possessed highly unique GCF profiles compared to other *Alternaria* sections, providing nine ideal candidates for diagnostic or chemotaxonomic marker development. However, none of these GCFs were associated with known compounds, prompting further research into the genetic characterization of *Alternaria* SMs. The GCF for the most prominent *Alternaria* mycotoxin alternariol (AOH) was found in *Alternaria* sections *Alternaria* and *Porri*, suggesting that food safety monitoring efforts should prioritize these two sections. Findings supported phytosanitary regulations regarding *Alternaria gaisen*, associated with Asian pear host-specific AK-toxin I.

**Conclusions:**

Our analyses are of unprecedented scale and resolution, allowing the identification of SM BGCs that are shared among multiple genera, or restricted to certain groups of focal taxa. Our study highlights the challenges associated with exploratory genome mining as a launching point for further research, and informs *Alternaria* disease management and regulation, food safety practices, and natural product discovery.

**Supplementary Information:**

The online version contains supplementary material available at 10.1186/s12864-025-11754-z.

## Background

Microbes produce a wide range of secondary metabolites (SMs) – small, specialized, bioactive molecules that are not essential to survival, but may provide a competitive advantage to the organisms that produce them under certain environmental conditions. SMs have diverse functions and chemical structures that have been critical in the development of important industrial compounds [1]. SM discovery was initially driven by a “top-down” approach involving phenotypic screening of the bioactive natural products in fermentation broths [2]. This approach quickly lost momentum due to a high rate of rediscovery and low throughput stemming from insufficiently sensitive technology [2]. The advancement of high-throughput sequencing and bioinformatics tools has since facilitated a new discovery approach: genome mining. Genome mining was initially applied to single genomes at a time, but with improvements to in silico approaches and decreasing sequencing costs, the number of whole genomes available for study grew rapidly. Now, in silico approaches are able to perform large-scale analyses on taxonomically diverse organisms for systematic exploration of their potential to produce SMs [3].

SM genome mining relies on the fact that SM biosynthesis usually requires at least two enzymes whose associated genes are situated in close proximity to each other on the chromosome, in structures called biosynthetic gene clusters (BGCs). The collection of BGCs in an organism’s genome reflects its potential to produce SMs. BGCs include one or two main biosynthetic enzymes, known as backbone genes, which determine the class of SM produced. Other genes in the cluster may be tailoring enzymes that act on intermediates and/or modify the final product into derived SMs: transcription factors that regulate expression of one or all genes in the cluster, or transport proteins that localize biosynthetic reactions or carry products elsewhere. Differences in tailoring enzymes may result in some structural variation although BGCs that share a “cluster core” are predicted to produce highly similar SMs, as corroborated by mass spectrometry studies [4, 5]. BGC genes can be readily identified in a genome sequence and annotated by consulting databases such as MIBiG (Minimum Information about a Biosynthetic Gene cluster) [6]. The complexity and diversity in gene cluster architecture can be studied to identify taxon-specific patterns and build BGC profiles that inform food safety standards, toxicological risk assessments, and trade regulations related to fungi of concern.

Here, we present the first large-scale genome-mining study for the fungal genus *Alternaria* and close relatives, highlighting the research potential of this type of exploratory analysis. *Alternaria* species are some of the most frequently recovered fungi in a wide range of environments [7–9] and many have significant impacts on agricultural crops and food production. The genus can be divided into 27 sub-generic sections, among which *Alternaria* section *Alternaria* is of particular agricultural concern. Some strains from section *Alternaria* have been sorted into sub-species categories (*formae speciales*, pathotypes) according to their ability to produce host-specific toxins (HSTs), suggestive of distinct mycotoxin profiles [10–14]. For instance, AK-toxin I is thought to be the compound allowing the pear pathotype of *A. gaisen* to infect the Asian pear [15]. Sections *Infectoriae* and *Pseudoalternaria* (*Inf/Pse*) are more distantly related to other *Alternaria* sections regarding phylogeny, morphology, and biochemistry, but are closely related to each other [16–19]. Continuing metabolomics efforts have identified over 260 *Alternaria* SMs with different chemical structures and behaviors [20]. The diversity of these SMs reflects *Alternaria*’s wide host range. Some SMs have received attention for their beneficial antimicrobial and pharmaceutical properties, and others for their concern as emerging phytotoxins and cytotoxins [21]. There are currently more than 70 characterized toxins that may cause problems both pre- and post-harvest, but there are no regulations in place to monitor or control them [21, 22]. The most prevalent genotoxic *Alternaria* food toxins, alternariol (AOH) and alternariol monomethyl ether (AME), have been observed to accumulate in crops and various processed food products at levels exceeding the threshold of toxicological concern [11, 22, 23]. In vitro studies suggest that AOH and AME exhibit cytotoxic, fetotoxic, and genotoxic effects in human and animal cell cultures, highlighting the need to manage these toxins [22–24].

Despite the health hazards that *Alternaria* toxins pose, a thorough risk assessment is yet to be performed. Toxicity data are scarce and the genetics behind their biosynthesis are not well understood, due in part to the lack of sampling across *Alternaria*’s full range of biodiversity. To address this issue, we explored the metabolic potential of this genus by mining BGCs from 123 whole genome assemblies from nine *Alternaria* sections. Our dataset includes a conservative estimate of 28 different *Alternaria* species. Thirty-seven newly generated *Alternaria* genomes are reported here, including those from three previously unrepresented *Alternaria* sections. While our main focus is the distribution of BGCs across *Alternaria*, we also analyzed 64 strains from seven closely related genera within the family Pleosporaceae, containing a large number of economically important plant pathogenic fungi. This broader sampling of the family allowed the identification of BGCs that are shared among multiple genera or restricted to certain groups of taxa, such as *Alternaria*-specific BGCs. BGC distribution patterns were correlated with phylogenomic patterns and the presence/absence of particular BGCs of interest (e.g. AK-toxin I, AOH, and AME), revealing insight into how the results of this exploratory research could be used to direct future targeted studies. Our study highlights the challenges associated with exploratory genome mining as a launching point for further research, and informs *Alternaria* disease management and regulation, food safety practices, and natural product discovery.

## Materials and methods

### Genomic resources and genome sequencing

A total of 187 genome assemblies were included in this study. Previously released *Alternaria* genomes were downloaded from the National Centre for Biotechnology Information (NCBI) database and the Joint Genome Institute (JGI) genome portal on July 28, 2021 by searching "Alternaria"[Organism] AND"ascomycetes"[orgn]. In cases where multiple genome versions existed, the most recently uploaded version was used. Assembly quality was checked with QUAST (QUality ASsessment Tool) ver. 5.0.2 [25]. Genomes with inconsistent genome size (> 50 Mb) or a high percentage of uncalled bases (> 1.5% Ns) were omitted, with 64 *Alternaria* genomes being retained for analyses. The same process was repeated for all other genera in the family Pleosporaceae; August 20, 2021 with search term (("Pleosporaceae"[Organism]) NOT "Alternaria"[Organism]) AND "ascomycetes"[orgn]. As above, duplicated and low quality accessions were omitted, with 64 “other” Pleosporaceae (non-*Alternaria*) genomes being retained. Resolution of nomenclatural inconsistencies is described in Supplementary Material.

We generated whole-genome sequences for an additional 59 *Alternaria* strains to support this study. Twenty-two of these genomes have been published [16, 26, 27] and the remaining 37 unpublished *Alternaria* genomes are being released here. For the 37 newly released genomes, all but one strain (KAS1274) were collected from within Canada. These strains were isolated from the urban environment or from one of 15 different plant host genera, including common agricultural crops such as wheat, barley, oats, canola, tomato, carrot, and apple. The methods used for DNA extraction, sequencing, and assembly are outlined in Dettman and Eggertson 2021 [26]. Briefly, sequencing was performed with a NextSeq500 instrument (Illumina, San Diego, California, USA), raw reads were clipped with Trimmomatic ver. 0.38 [28] and de novo assembly was done with SPAdes (St. Petersburg genome Assembler) ver. 3.12 [29]. All assemblies, accession numbers, and other relevant statistics are listed in Table S1, Supplementary Material.

### Gene prediction using the funannotate pipeline

Gene prediction is required to identify SM-related BGCs downstream; however, the included assemblies were from various sources and in different states of annotation. To remove bias that may be caused by technical variation between analysis pipelines, gene prediction and annotation were re-performed on all assemblies using a single, unified methodology. The funannotate pipeline (ver. 1.8.7; https://github.com/nextgenusfs/funannotate) is a lightweight platform that is designed to perform genome masking, gene prediction, and functional annotation of fungal genomes. It incorporates a number of well-established tools and integrates several gene prediction and annotation approaches, providing an advantage over other annotation pipelines. A list of related programs and versions can be found in Table S2, Supplementary Material. It is important to note that gene prediction can be performed in a multitude of ways, depending on gene calling algorithms, forms of evidence, weighting of evidence, etc. To ensure we were using the optimized methods for gene prediction across our phylogenetically diverse genome sample, we performed a series of runs for parameter testing and results comparison, summarized below (Table S3, Supplementary Material). Assemblies were first cleaned and masked on the default settings. The < clean > command removes small duplicated contigs while < mask > softmasks short tandem repeats and low complexity repeats with tantan. FASTA headers were renamed while retaining the original scaffold order. Custom training parameters were generated for *Alternaria* genomes by providing genomic and transcriptomic data from *A. alternata* strain SRC1lrK2f (NCBI accession: SRR4063372) to the < train > command. Briefly, RNA sequences were assembled using Trinity, while PASA modeled gene structures based on transcription alignments. These processes created a set of parameter files that can be saved and evoked later to train and run Augustus, glimmerHMM, and snap during the < predict > step. Gene predictors for non-*Alternaria* genomes used general Augustus parameters pre-trained on *Aspergillus nidulans*. Of the taxa for which pre-trained parameters are available, *A. nidulans* is the most closely related to the Pleosporaceae family, but is also distant enough that gene counts would not be biased for certain genera. In the predict step, a set of pre-downloaded proteins from the UniProt/Swiss-Prot database were used to guide funannotate’s suite of gene predictors, including GeneMark-ES, Augustus, snap, and glimmerHMM during the generation of several sets of gene models. Evidence from all predictors was passed to EvidenceModeler which combined gene models into a weighted final set.

### BGC identification and annotation

After gene prediction, BGC identification was performed with antiSMASH (antibiotics & Secondary Metabolite Analysis Shell) ver. 6.0 for fungi [30], with all options turned on. BGCs identified by antiSMASH were clustered using BiG-SCAPE (Biosynthetic Gene Similarity Clustering and Prospecting Engine) ver. 1.1.2 [31] with the following flags: < –include_singletons –hybrids-off –cutoffs 0.4 –clans-off –mode global >. The < –hybrids-off > flag was invoked to prevent duplication of BGCs into multiple classes and artificially inflate BGC counts [31]. The resulting interaction network files were visualized with Cytoscape ver. 3.10.3 [32]. Similar BGCs were grouped into higher-level gene cluster families (GCFs) using the MCL clustering algorithm available in the AutoAnnotate application ver. 1.5.1 (https://autoannotate.readthedocs.io/). GCF membership data were used to generate presence/absence tile plots in RStudio ver. 1.1.463 to display the distribution of GCFs across the phylogeny. BGCs were compared and visualized with clinker v0.0.30 [33] in which amino acid sequences are extracted from input BGCs and compared in all-vs-all global alignments. Cluster similarity scores, incorporating both sequence similarity and syntenic conservation, were calculated for all pairs of inputs.

### Phylogenetic reconstruction

The organismal phylogeny was estimated by OrthoFinder ver. 2.5.4 [34] by using the concatenated MAFFT alignment (-M msa) of shared, single-copy orthogroups. A maximum likelihood tree was inferred with FastTree and local branch support values were calculated with a SH-like (Shimodaira-Hasegawa) test, with values > 95 considered significant [35–37]. *Decorospora* was chosen as the outgroup as suggested by the tree topologies reported in Haridas et al. 2020 [38].

## Results

### Taxonomic composition of genomic dataset

For a more holistic approach to studying BGC distributions, the genus *Alternaria* and close relatives within Pleosporaceae were analyzed simultaneously, with the goal of capturing the maximum amount of this fungal family’s genetic diversity as possible. For *Alternaria*, the dataset included 123 genome assemblies: 86 publicly available and 37 novel, unpublished assemblies. Our newly generated genomes expanded the coverage of sections *Alternaria*, *Infectoriae*, *Pseudoalternaria*, and *Ulocladioides*, and provided the first genomes from sections *Embellisia*, *Ulocladium*, and *Pseudoulocladium*. To help observe patterns at a higher taxonomic level, *Alternaria* strains were placed into three groups in our analyses:

1. *Alternaria* section *Alternaria*, containing 70% of the *Alternaria* genomes.
2. *Alternaria* sections *Infectoriae* and *Pseudoalternaria* (*Inf/Pse*). These two sections are closely related and are predicted to have similar SM profiles.
3. The six remaining *Alternaria* sections and the one monotypic lineage, collectively referred to as “other” *Alternaria*. The scope of genetic diversity in this group is quite large, but the sample sizes for separate sections were too limited to analyze individually.

For “other” Pleosporaceae (non-*Alternaria*), 64 genomes from seven genera (*Bipolaris*, *Curvularia*, *Decorospora*, *Paradendryphiella*, *Pyrenophora*, *Setosphaeria*, and *Stemphylium*) were included to allow the *Alternaria* results to be placed in a fuller taxonomic context. The final dataset used in this study included 187 whole genomes (Table S1, Table S4, Supplementary Material).

### Gene model prediction

Gene model prediction was performed on all genomes, resulting in 9,168–13,505 gene models per assembly (Table S1, Supplementary Material). Other Pleosporaceae genera, especially *Pyrenophora*, typically had lower gene model counts (< 12 k) compared to *Alternaria*. This result may be expected because gene predictors for *Alternaria* genomes were optimized on section *Alternaria* RNA-seq data (Supplementary Material). Gene prediction for non-*Alternaria* genomes was performed ab initio, without genus-specific training, so the same level of accuracy may not have been reached. Among *Alternaria* sections, section *Brassicicola* had the lowest gene counts that were comparable to “other” Pleosporaceae genomes. Sections *Porri* and *Ulocladioides* also had relatively low gene counts, averaging 11.3 k and 11.9 k genes, respectively. Sections *Alternaria* and *Inf/Pse* tended to have the highest gene counts, both averaging 12.3 k genes. This trend was not expected because sections *Inf/Pse* are early-diverging and phylogenetically distinct from section *Alternaria*, and the benefits of gene predictor training are expected to transfer to more closely related rather than distantly related taxa. These results suggest that these sections have more similar gene structures (e.g. intron/exon boundaries), or that strains in these sections simply have more genes.

### Identification and annotation of all SM BGCs

**Fig. 1:**
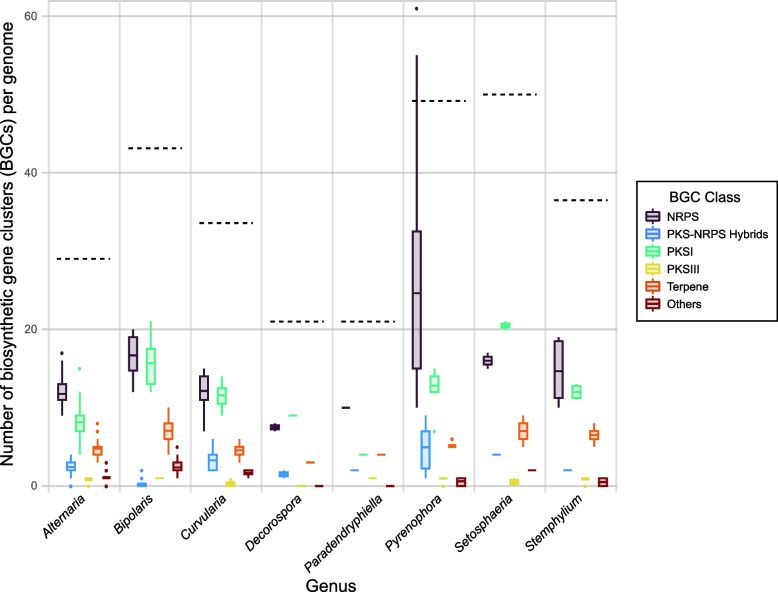
Boxplot distributions of the number of BGCs per genome, by BGC class and by genus. Dotted line represents the mean total number of BGCs per genus. For specific BGC classes, the solid middle line represents the mean; boxes represent the first and third interquartile ranges (IQR); extreme lines extend 1.5 × IQR above and below the box; dots are potential outliers

Next, antiSMASH was used to identify SM BGCs in all 187 annotated genomes. There were 21–85 BGCs (mean = 34.0) detected per genome (Table S1, Supplementary Material), resulting in a cumulative total of 6,323 BGCs across all genomes. *Alternaria* genomes encoded 21–40 BGCs, with an average of 29.0 per genome (Fig. 1). Non-*Alternaria* Pleosporaceae strains tended to have more BGCs compared to *Alternaria* strains, with an average of 42.0 per genome. *Pyrenophora* and *Setosphaeria* genomes had the highest BGC counts.

BGCs were grouped into six classes that correspond to the encoded SM product or how it is produced: NRPS (non-ribosomal peptide synthetase), PKSI (type 1 polyketide synthase), PKS-NRPS Hybrids, PKSIII (type 3 polyketide synthase), Terpene, and Others (Table S5, Supplementary Material). Each genome had 7–61 NRPS, 1–9 PKS-NRPS Hybrids, 4–21 PKSI, 0–1 PKSIII, 3–10 Terpene, and 1–5 Other BGCs. Specifically, *Alternaria* genomes had 9–17 NRPS, 1–4 PKS-NRPS Hybrids, 4–15 PKSI, 0–1 PKSIII, 3–8 Terpene, and 1–3 Other BGCs. A boxplot summarizing the distributions and counts of each BGC class, by genus, was generated (Fig. 1). In general, the relative distribution of BGCs across BGC classes was similar across genera. Notably, the number and variance in NRPS gene clusters per strain for *Pyrenophora* was higher than in any other class or genus. Other exceptions were that *Setosphaeria* tended to have more PKSI BGCs, and *Bipolaris* had comparatively less PKS-NRPS hybrid BGCs. The small sample size of certain genera (*Decorospora*, *Paradendryphiella*, and *Setosphaeria*) should be acknowledged (Table S4, Supplementary Material).

### Clustering of BGCs into GCFs

BiG-SCAPE [31] was used to calculate sequence similarity, protein domain content, and protein domain synteny between all BGCs based on a combination of three distance matrices (Jaccard index, Adjacency index, Domain Sequence Identity). Similarity networks were generated and BGCs connected by edges were grouped into a GCF. A total of 548 GCFs (Table 1) were identified, with the vast majority (~ 75.2%) falling within the NRPS or PKSI classes, and with each genome containing an average of 12.75 NRPS and 9.75 PKSI GCFs. The average number of total GCFs per genome was 31.81, and the average prevalence of a GCF across all genomes was only 6.0%. Furthermore, 54.0% of GCFs were unique to a single genome (singletons), demonstrating high levels of non-redundant SM BGC diversity present in these fungi.

**Table 1:** Numbers of identified BGCs and GCFs

	Number of BGCs	Number of GCFs	Average number of GCFs per genome	Average prevalence of GCFs (%)	Percentage of GCFs unique to one genome (singletons)
NRPS	2624	259	12.75	4.92	64.48
PKSI	1841	153	9.75	6.37	38.56
PKS-NRPS Hybrids	543	72	2.97	3.99	54.17
PKSOther	149	8	1.00	9.96	50.00
RiPPs	110	3	1.00	19.61	0.00
Terpene	946	53	5.05	9.53	50.94

### Predicted compounds produced by GCFs

antiSMASH compares each BGC to known BGCs in the annotated MIBiG database to generate predictions for associated compounds. To determine representative compounds for each GCF, all predicted BGC annotations were extracted, compiled, and manually curated. Many predicted annotations were rejected after critical comparison of the MIBiG entry with the structure of the query BGCs, taking into account the similarity of BGC structures (e.g. number of matching genes) and sequence similarity of important genes. Altogether, we accepted the compound predictions for 28 GCFs, which were associated with 19 different compounds (Table 2.).

**Table 2:** Prevalence of GCFs with associated compounds, across 187 taxa divided into four groups

GCF class	GCF #	% *Alternaria* section *Alternaria* (n=86)	% *Alternaria* section *Infectoriae/ Pseudoalternaria* (n=15)	% "other" *Alternaria* (n=22)	% "other" Pleosporaceae (n=64)	Predicted compound, MiBIG accession
**PKS-NRPS Hybrids**	**23**	**3.5**	**0.0**	**0.0**	**0.0**	**ACT-Toxin II, BGC0001254**
PKSI	35	7.0	0.0	0.0	6.3	AK-toxin, BGC0001262;AF-toxin, BGC0000003
PKSI	3	100.0	100.0	50.0	73.4	alternapyrone, BGC0000012
**PKSI**	**5**	**97.7**	**0.0**	**45.5**	**0.0**	**alternariol, BGC0001284**
**NRPS**	**37**	**7.0**	**0.0**	**0.0**	**0.0**	**AM-toxin, BGC0001261**
NRPS	13	0.0	0.0	0.0	46.9	apicidin, BGC0000304
PKSI	6	47.7	0.0	13.6	64.1	betaenone A, BGC0001264
PKS-NRPS Hybrids	17	0.0	0.0	22.7	1.6	BII-rafflesfungin, BGC0001966
**Terpene**	**16**	**0.0**	**0.0**	**13.6**	**0.0**	**brassicicene C, BGC0000685**
PKS-NRPS Hybrids	9	0.0	0.0	0.0	25.0	curvupallide-B, BGC0001563
PKS-NRPS Hybrids	60	0.0	0.0	0.0	1.6	curvupallide-B, BGC0001563
PKSI	16	3.5	0.0	45.5	18.8	dehydrocurvularin, BGC0000045
**PKSI**	**67**	**0.0**	**0.0**	**13.6**	**0.0**	**depudecin, BGC0000046**
NRPS	6	100.0	100.0	86.4	89.1	dimethylcoprogen, BGC0001249
PKS-NRPS Hybrids	21	0.0	0.0	0.0	7.8	dimethylcoprogen, BGC0001249
**PKS-NRPS Hybrids**	**25**	**0.0**	**0.0**	**13.6**	**0.0**	dimethylcoprogen, BGC0001249
PKS-NRPS Hybrids	31	0.0	0.0	0.0	3.1	dimethylcoprogen, BGC0001249
**NRPS**	**76**	**2.3**	**0.0**	**0.0**	**0.0**	**altersetin/equisetin, BGC0001255**
**NRPS**	**99**	**1.2**	**0.0**	**0.0**	**0.0**	**altersetin/equisetin, BGC0001255**
**PKS-NRPS Hybrids**	**2**	**84.9**	**0.0**	**0.0**	**0.0**	**altersetin/equisetin, BGC0001255**
NRPS	63	0.0	0.0	4.5	1.6	HC-toxin, BGC0001166
**NRPS**	**86**	**0.0**	**0.0**	**9.1**	**0.0**	**KK-1, BGC0001636**
PKSI	1	98.8	100.0	90.9	100.0	melanin, BGC0001265
NRPS	169	0.0	0.0	0.0	1.6	phomasetin, BGC0001738
NRPS	185	0.0	0.0	0.0	1.6	phomasetin, BGC0001738
PKS-NRPS Hybrids	4	0.0	0.0	13.6	28.1	phomasetin, BGC0001738
**PKSI**	**58**	**3.5**	**0.0**	**4.5**	**0.0**	**solanapyrone D, BGC0000146**
**PKSI**	**90**	**0.0**	**0.0**	**9.1**	**0.0**	**solanapyrone D, BGC0000146**

Bolded rows indicate GCFs found in only *Alternaria* genomes

### Taxonomic composition of GCF membership

**Fig. 2:**
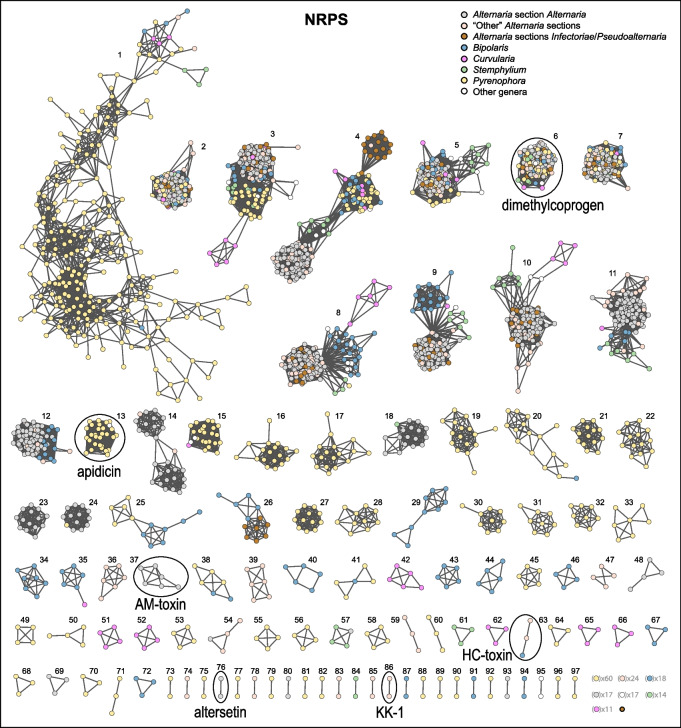
GCF networks for the NRPS class. GCFs are numbered according to the named GCFs and displayed in descending order of network size. Each node represents a BGC and lines (edges) connect nodes with distances less than or equal to the cutoff threshold (0.4). Nodes are color-coded by taxonomic group, as shown in the legend. Singletons are displayed as a representative single node, with the number of such singletons indicated beside each single node. GCFs associated with known compounds are circled and annotated

**Fig. 3:**
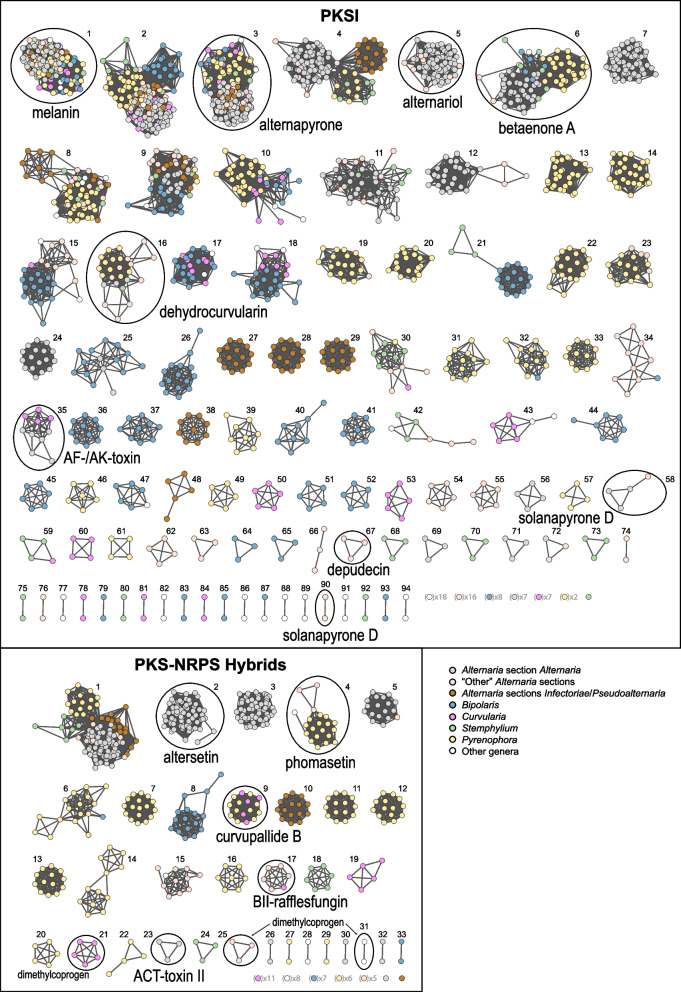
GCF networks for the PKSI and PKS-NRPS hybrid classes. GCFs are numbered according to the named GCFs and displayed in descending order of network size. Each node represents a BGC and lines (edges) connect nodes with distances less than or equal to the cutoff threshold (0.4). Nodes are color-coded by taxonomic group, as shown in the legend. Singletons are displayed as a representative single node, with the number of such singletons indicated beside each single node. GCFs associated with known compounds are circled and annotated

To assess the taxonomic membership of GCFs, network diagrams were visualized and nodes were colour-coded by taxa (Figs. 2 and 3 for NRPS, PKSI, and PKS-NRPS Hybrids; Fig. S1, Supplementary Material for Terpene, PKSOther, and RiPPs). In general, larger GCFs tended to have broader distributions across taxa, whereas smaller GCFs tended to have more restricted or taxonomically localized membership. An exception to this pattern was NRPS 1 (Fig. 2), which had the largest number of members at 231 BGCs, but relatively low overall prevalence, being found in only 26 genomes (13.9%). Of these, half were *P. teres* genomes containing multiple putatively duplicated copies of BGCs in this GCF. Some *P. teres* genomes possessed up to 42 copies, averaging 16.8 per genome. The network for NRPS 1 was asymmetrical and intransitive, suggesting that while the BGCs are similar enough to be included in the same GCF, some are also notably dissimilar. GCFs with the most taxonomic heterogeneity included NRPS 2, NRPS 6, NRPS 7, PKSI 1, Terpene 1, Terpene 2, and Terpene 3. These GCF networks tended to be tightly bunched with little taxonomic correlation.

**Fig. 4:**
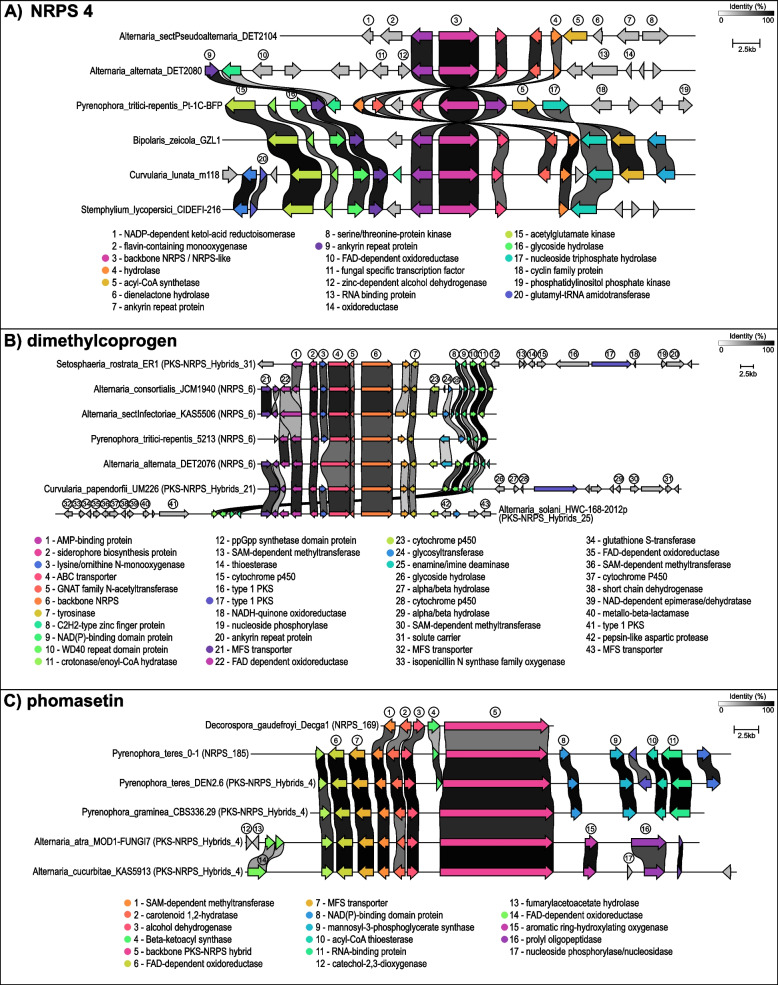
Comparison of representative BGCs from **A** NRPS 4, **B** dimethylcoprogen-associated GCFs, and **C** phomasetin-associated GCFs. Within each panel, homologous genes in adjacently displayed BGCs are linked with percent identity indicated by a quantitative scale (white to black, see legend). Homologous genes are also color coded. Genes shown in grey do not have identified homologs in the other BGCs. Genes with relevant annotations are numbered and their predicted functions are listed below the gene cluster diagram

In contrast, some GCFs contained observable BGC sub-groups with taxa-specific distributional patterns. In NRPS 4, for example, BGCs from genomes in *Alternaria* sections *Inf/Pse* formed a sub-group distinct from the other *Alternaria* groups, as did *Stemphylium* from the other genera, although not exclusively (Fig. 2). A comparison of representative BGCs from NRPS 4 illustrated the gene structure differences likely accounting for the taxa-specific sub-groups (Fig. 4A). While the backbone gene is shared among all taxa, slight differences in levels of sequence divergence and the presence/absence of specific accessory genes both contribute to the observed sub-grouping patterns. Other GCFs with discernable genus-specific sub-grouping include NRPS 8, NRPS 9, NRPS 10, PKSI 2, and PKSI 4, demonstrating BGC diversity within GCFs and between taxa.

Interestingly, only three RiPP GCFs were identified and detected in only 110 strains from three genera (*Alternaria*, *Curvularia*, and *Bipolaris*). Two of the three RiPP GCFs were found only in *Alternaria*, and 92.7% of all RiPP BGCs were from *Alternaria*, demonstrating a clear taxonomic bias for this class.

### Distribution of GCFs across the organismal phylogeny

**Fig. 5:**
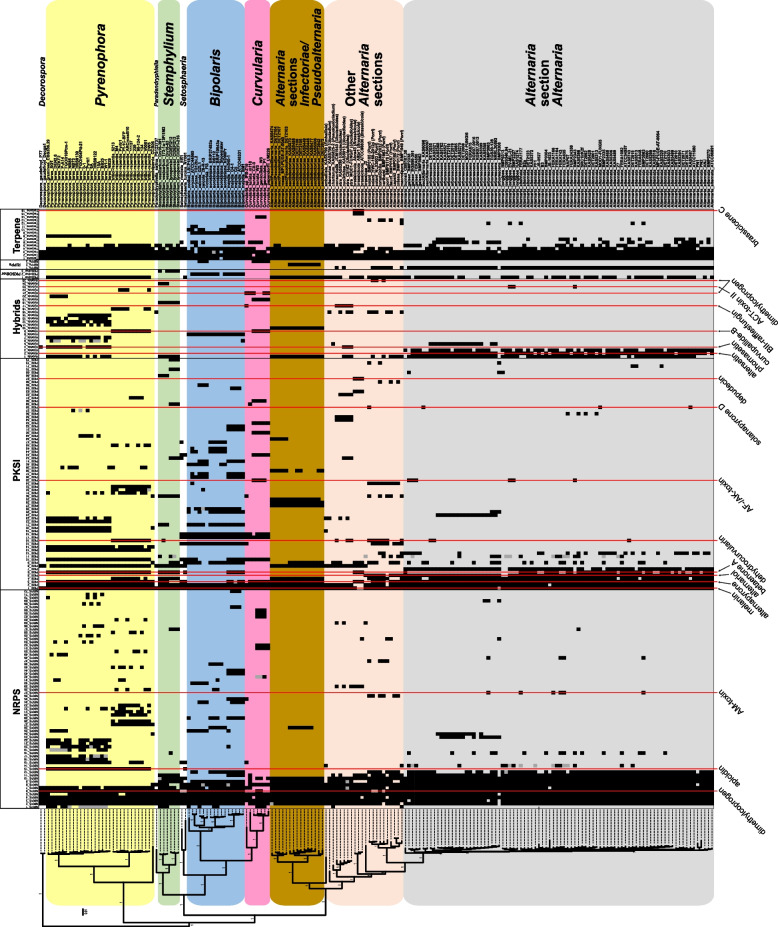
Distribution of GCFs (x-axis) in dataset ordered by the organismal phylogeny (y axis). Phylogeny is based on 1,101 shared single-copy orthologs from 187 Pleosporaceae genomes. Only GCFs present in at least three genomes are shown (prevalence of all GCFs can be found in Table S6, Supplementary Material). Numbers on main branches of phylogeny indicate Shimodaira-Hasegawa-like branch support (all = 1.0). Filled black squares indicate presence of the GCF in the respective genome. Grey squares indicate that multiple BGCs from the respective genome are within that GCF. *Alternaria* sub-groups and genera with more than two genomes are colour-coded by horizontal blocks. Vertical red lines track along GCFs with annotations to compounds, listed along the bottom

A maximum likelihood phylogenetic tree of all 187 taxa was created with OrthoFinder [34] using protein sequences from 1,101 shared, single-copy orthogroups (Fig. 5). As a result of the large number of orthogroups included, all main branches were very well supported (SH-like supports > 95). The relationships within the genus *Alternaria* were concordant with previous studies [14, 26, 39]. The nine *Alternaria* sections (*Alternaria*, *Brassicicola*, *Embellisia*, *Infectoriae*, *Porri*, *Pseudoalternaria*, *Pseudoulocladium*, *Ulocladioides*, *Ulocladium*) were well resolved and distinct from each other. Within section *Alternaria*, the four main lineages were identified, as previously observed [26], including the alternata, gaisen, longipes, and arborescens lineages. The relationships between genera within Pleosporaceae were consistent with previous studies [38, 40]. Therefore, the tree was considered to be a reliable estimation of the evolutionary relationships among the taxa.

To visualize the distribution of GCFs across the organismal phylogeny, GCFs present in at least 3 taxa were plotted in a presence/absence heatmap and superimposed back onto the reference phylogeny produced from phylogenomic data (Fig. 5). Overall, the distribution of the GCFs appeared to be fairly consistent with phylogenetic patterns, such that more closely related taxa had more similar GCF presence/absence patterns. While a wide range of GCF distribution patterns were observed, here we focus on three that are most relevant to this study: 1) widely distributed, 2) present only in “other” Pleosporaceae (non-*Alternaria*) genomes, and 3) present only in *Alternaria* genomes.

#### Widely distributed GCFs and associated compounds

**Fig. 6:**
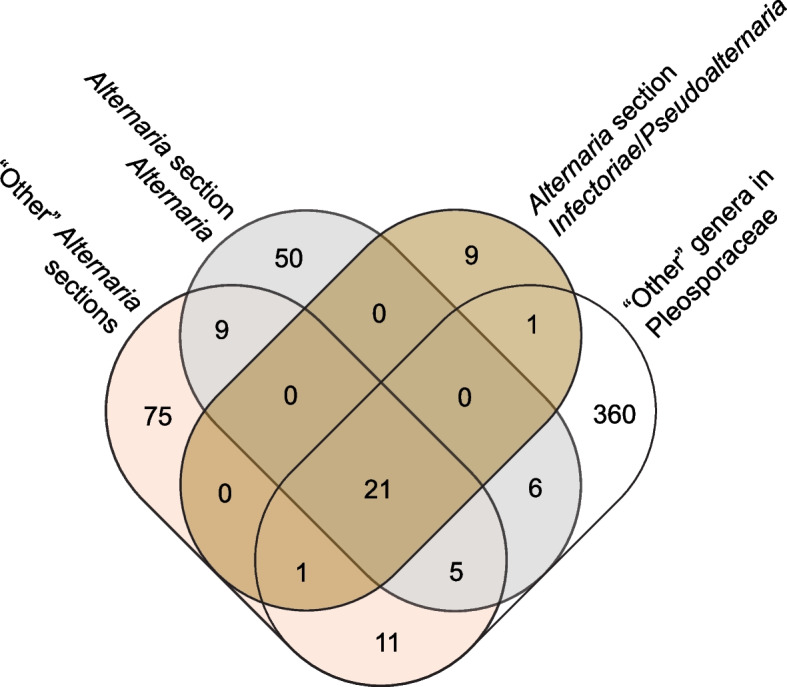
Venn diagram showing the number of GCFs unique to, or shared by, specific taxonomic groups. Results are shown for all 548 GCFs with the six GCF classes combined (NPRS, PKSI, PKS-NRPS Hybrids, PKSOther, Terpenes, and RiPPs)

Overall, only 45 of the 548 (8.2%) GCFs were shared between *Alternaria* and “other” Pleosporaceae genomes (Fig. 6; 17 NRPS, 17 PKSI, 6 Terpene, 4 PKS-NRPS Hybrids, 1 PKSOther). There were 11 GCFs with > 90% overall prevalence (Table S6, Supplementary Material), suggesting that some SMs and related compounds may be produced by most Pleosporaceae fungi. Of the 11 widely-distributed GCFs, only two (PKSI 1 and NRPS 6) were associated with known compounds. PKSI 1 was linked to melanin (MIBiG: BGC0001265), an abundant natural pigment known to increase virulence of many pathogenic fungi and is ubiquitously synthesized by fungi, bacteria, and other microorganisms [41–43]. PKSI 1 was missing from three *Alternaria* strains (*A. arborescens* BMP0308, *A. brassicicola* Altbr1, *A. brassicicola* ATCC96836). All genomes in this dataset are expected to be able to produce melanin; failure to detect PKSI 1 is likely due to errors in gene prediction and/or BGC identification. NRPS 6 was associated with dimethylcoprogen (MIBiG: BGC0001249, Fig. 4B), a siderophore that allows fungi and other microorganisms to effectively uptake iron from their environment [44–46]. Although the dimethylcoprogen GCF was expected to be found in all taxa, NRPS 6 was missing from 10 genomes—three from *Alternaria* section *Porri*, five from *Curvularia*, and two from *Setosphaeria* (Fig. 5). Closer examination of GCF annotations revealed some others were also associated with dimethylcoprogen, and that these GCFs, named as PKS-NRPS Hybrids 21, PKS-NRPS Hybrids 25, and PKS-NRPS Hybrids 31, were found in those exact missing strains from *Curvularia*, *Alternaria*, and *Setosphaeria*, respectively. In total, all 187 genomes were found to contain at least one dimethylcoprogen-related BGC.

Seven other GCFs shared between *Alternaria* and “other” Pleosporaceae were linked to known compounds (Table 2.): alternapyrone (PKSI 3; 159 genomes; MIBiG: BGC0000012), betaenone A (PKSI 6; 85 genomes; MIBiG: BGC0001264, renamed BGC0001280), dehydrocurvularin (PKSI 16; 25 genomes; MIBiG: BGC0000045), phomasetin (PKS-NRPS Hybrids 4; 21 genomes; MIBiG: BGC0001738), AK-toxin or AF-toxin (PKSI 35; 10 genomes; MIBiG: BGC0001262), BII-rafflesfungin (PKS-NRPS Hybrids 17; 6 genomes; MIBiG: BGC0001966), and HC-toxin (NRPS 63; 3 genomes; MIBiG: BGC0001166). Host-specific toxins (HSTs) associated with *Alternaria* will be described in later sections.

Alternapyrone and betaenone A were linked to PKSI 3 and PKSI 6, respectively, both of which were found fairly commonly in at least five of the sampled genera. Dehydrocurvularin-linked PKSI 16 had a more scattered distribution, and was found only in *Alternaria* sections *Alternaria* (3/86)*, Porri* (7/10), and *Ulocladioides* (3/5), *P. tritici-repentis* (11/11), and *Stemphylium* (1/6). PKS-NRPS Hybrids 17, associated with BII-rafflesfungin, was found only in *Alternaria* section *Ulocladioides* (5/5) and one *Curvularia* genome (1/7).

The compound phomasetin was also associated with multiple different GCFs (Fig. 4C), including NRPS 169 (1/2 *Decorospora)*, NRPS 185 (1/17 *P. teres*), and PKS-NRPS Hybrids 4 (3/4 *Alternaria* section *Ulocladioides*, 1/2 *Decorospora*, 16/17 *P. teres*, 1/1 *P. graminea*). Combining the three GCFs, all *Decorospora* and *P. teres* genomes were predicted to encode a phomasetin BGC. Phomasetin was originally discovered in *Phoma* species as a stereochemical homolog of equisetin and is reported to have a wide range of biological activity [47].

#### GCFs present only in “other” Pleosporaceae (non-Alternaria) genomes

There were 360 GCFs found only in “other” Pleosporaceae strains: 182 NRPS, 90 PKSI, 52 PKS-NRPS Hybrids, 29 Terpene, 6 PKSOther, 1 RiPPs (Fig. 6). Seven of these GCFs were linked to four named compounds, two of which were unique to non-*Alternaria* genomes: apicidin (NRPS 13; MIBiG: BGC0000304) and curvupallide-B (PKS-NRPS Hybrids 9 and PKS-NRPS Hybrids 60; MIBiG: BGC0001563).

NRPS 13, related to apicidin, was the second most prevalent among non-*Alternaria* genomes (46.9%), found in 29/30 *Pyrenophora* and 1/2 *Setosphaeria* genomes. Apicidin, a cell-permeable histone deacetylase inhibitor, is a potent antiparasitic fungal metabolite that also demonstrates antiproliferative activity in some lines of tumour cells [48]. GCFs for curvupallide-B, first described from the phytopathogen *C. pallescens* [49], were recovered from 5/7 *Curvularia*, 11/11 *P. tritici-repentis*, and 1/1 *P. seminiperda* genomes. Curvupallides do not exhibit phytotoxic activity, although a structurally similar class of compounds, spirostaphylotrichines, do [49]. The remaining GCFs did not match significantly to known SMs.

PKSI 10 was the most prevalent GCF and was found in 71.9% of “other” Pleosporaceae genomes: *Pyrenophora* (29/30), *Bipolaris* (8/16), *Curvularia* (7/7), and *Setosphaeria* (2/2). The remainder of GCFs had < 47% prevalence and tended to have genus-specific distributions, occasionally with rare hits from another genus. For instance, the third and fourth most prevalent “other” Pleosporaceae-specific GCFs, PKSI 13 and PKSI 14 (45.3% and 43.8%, respectively) were found only in *Pyrenophora* genomes.

Interestingly, 213 (59.2%) of the 360 GCFs were singletons, represented by only one genome. There were 73 singletons in *Pyrenophora*, 41 in *Bipolaris*, 39 in *Setosphaeria*, 30 in *Curvularia*, 21 in *Stemphylium*, eight in *Paradendryphiella,* and one in *Decorospora*.

#### GCFs present only in Alternaria genomes

In total, 143 GCFs were found exclusively in the genus *Alternaria* (60 NRPS, 46 PKSI, 18 Terpene, 16 PKS-NRPS Hybrids, 2 RiPPs, 1 PKSOther; Fig. 6; Table 3). Only five of these GCFs were found with > 50% prevalence throughout the genus. The most prevalent, PKSI 5, was present in 94 genomes (84/86 section *Alternaria*, 9/10 section *Porri*, 1/1 monotypic lineage *A. brassicae*) and was associated with the compound AOH (MIBiG: BGC0001284). BLASTp searches of the backbone gene returned high-confidence matches at 96–100% identity to *pksI*, the non-reducing PKS responsible for the synthesis of AOH [50–52].

**Table 3:** GCFs recovered from *Alternaria* genomes only, with prevalence within *Alternaria* groups and their associated compounds

GCF class	GCF number	% sect. *Alternaria* (*n* = 86)	% sect. *Infectoriae/Pseudoalternaria* (*n* = 15)	% other *Alternaria* (*n* = 22)	Predicted compound, MiBIG accession
NRPS	14	20.9	0.0	9.1	
NRPS	23	16.3	0.0	0.0	
NRPS	36	0.0	0.0	27.3	
NRPS	37	7.0	0.0	0.0	AM-toxin, BGC0001261
NRPS	39	0.0	0.0	27.3	
NRPS	47	0.0	0.0	18.2	
NRPS	48	4.7	0.0	0.0	
NRPS	54	1.2	0.0	13.6	
NRPS	59	0.0	0.0	13.6	
NRPS	69	3.5	0.0	0.0	
PKS-NRPS Hybrids	2	84.9	0.0	0.0	altersetin/equisetin, BGC0001255
PKS-NRPS Hybrids	3	75.6	0.0	0.0	
PKS-NRPS Hybrids	5	19.8	0.0	4.5	
PKS-NRPS Hybrids	10	0.0	100.0	0.0	
PKS-NRPS Hybrids	23	3.5	0.0	0.0	ACT-Toxin II, BGC0001254
PKS-NRPS Hybrids	25	0.0	0.0	13.6	dimethylcoprogen, BGC0001249
PKSI	5	97.7	0.0	45.5	alternariol, BGC0001284
PKSI	7	90.7	0.0	0.0	
PKSI	12	32.6	0.0	13.6	
PKSI	24	19.8	0.0	0.0	
PKSI	27	0.0	100.0	0.0	
PKSI	28	0.0	100.0	0.0	
PKSI	29	0.0	93.3	0.0	
PKSI	38	0.0	60.0	0.0	
PKSI	48	0.0	33.3	0.0	
PKSI	54	0.0	0.0	22.7	
PKSI	55	0.0	0.0	22.7	
PKSI	56	4.7	0.0	0.0	
PKSI	58	3.5	0.0	4.5	solanapyrone D, BGC0000146
PKSI	62	0.0	0.0	18.2	
PKSI	63	0.0	0.0	13.6	
PKSI	66	0.0	0.0	13.6	
PKSI	67	0.0	0.0	13.6	depudecin, BGC0000046
PKSI	69	3.5	0.0	0.0	
PKSI	71	3.5	0.0	0.0	
PKSI	72	1.2	0.0	9.1	
RiPPs	1	97.7	0.0	40.9	
RiPPs	2	0.0	60.0	0.0	
Terpene	7	26.7	0.0	0.0	
Terpene	12	5.8	0.0	4.5	
Terpene	13	0.0	0.0	22.7	
Terpene	15	0.0	0.0	13.6	
Terpene	16	0.0	0.0	13.6	brassicicene C, BGC0000685

Only GCFs present in at least three genomes are shown. Prevalence of all GCFs can be found in Table S6, Supplementary Material

Among the 143 *Alternaria*-specific GCFs, 134 (93.7%) of them had even further restricted distributions, being present in only one of the three designated *Alternaria* sub-groups (Fig. 6). For example, 50 (35.0%) GCFs were found only in *Alternaria* section *Alternaria*, some with links to equisetin (MIBiG: BGC0001255) and HSTs such as ACT-toxin II (PKS-NRPS Hybrids 23; MIBiG: BGC0001254) and AM-toxin (NRPS 37; MIBiG: BGC0001261). Equisetin was associated with three different GCFs, collectively being found in 76 (88.4%) genomes from section *Alternaria*: NRPS 76 (n = 2), NRPS 99 (n = 1), and PKS-NRPS Hybrids 2 (n = 73). Links to equisetin were based on backbone gene matches of only 62% identity to the MIBiG entry from *Fusarium heterosporum* (Sordariomycetes, Hypocreales), a fairly distant relative to *Alternaria*. According to mass-spectrometry studies of the same strains, it is reasonable to hypothesize that these equisetin-related BGCs actually encode a structurally similar compound, altersetin [27, 53].

Seventy-five GCFs were restricted to “other” *Alternaria* sections, including those with BGCs predicted to produce brassicicene C (Terpene 16; MIBiG: BGC0000685), depudecin (PKSI 67; MIBiG: BGC0000046), dimethylcoprogen (PKS-NRPS Hybrids 25; MIBiG: BGC0001249), KK-1 (NRPS 86; MIBiG: BGC0001636), and solanapyrone D (PKSI 90; MIBiG: BGC0000146). Brassicicene C and depudecin were further restricted to section *Brassicicola* (3/3), and KK-1 was found in 1/1 section *Embellisia* and 1/5 section *Ulocladioides* genomes. Dimethylcoprogen and solanapyrone D were additionally linked to other GCFs that included genomes in both section *Alternaria* and “other” sections.

Nine GCFs were found exclusively in sections *Inf*/*Pse*; however, none were associated with known compounds. Interestingly, none of the genus-specific GCFs present in sections *Inf*/*Pse* were present in other *Alternaria* sections. GCFs PKSI 27, PKSI 28, and PKS-NRPS Hybrids 10 were found in all section *Inf*/*Pse* strains, providing good candidates for diagnostic or chemotaxonomic marker development.

### Distribution of GCFs associated with HSTs

Of the seven HSTs identified from *A. alternata* [13, 54], GCFs for ACT-toxin II, AF-toxin, AK-toxin, AM-toxin, and HC-toxin were predicted in strains of *Alternaria* section *Alternaria*, *Bipolaris*, and *Curvularia* (Table 2.). MIBiG entries are lacking for some other prominent *Alternaria* toxins such as the tomato HST, AAL-toxin, and the rough lemon HST, ACRL-toxin, and were not able to be identified in our dataset.

#### ACT-toxin

The tangerine HST ACT-toxin II GCF (MIBiG: BGC0001254; PKS-NRPS Hybrids 23) was restricted to three section *Alternaria* strains (*A. alternata* BMP2343, *A. longipes* BMP2327, *A. longipes* EV-MIL-31), all of which were isolated from citrus hosts. *A. alternata* BMP2343 and *A. longipes* BMP2327 were originally described as *A. citriarbusti* and *A. tangelonis*, respectively. These citrus-associated names refer to older naming conventions based on presumed host specificity that were used before Woudenberg et al. 2015 [14] synonymised many *Alternaria* morphospecies. BGCs had two backbone genes, one PKSI and one NRPS, resulting in their hybrid classification. The PKSI gene matched with 100% identity to the *A. alternata ACTTS3* gene, which produces the moiety characteristic of epoxy-decatrienoic acid (EDA) toxins. The NRPS gene had no credible matches in the MIBiG database, but BLASTp searches revealed high sequence identity (98.8%) with the *ACTTS4* gene (NRPS), one of the other five genes involved in ACT-toxin II biosynthesis [55–57]. All strains with the ACT-toxin II GCF also possessed PKSI 35, a different GCF which is predicted to encode for the production of related, EDA-based AF- and AK-toxins [13, 54, 58, 59].

#### AF-toxin and AK-toxin

The PKSI 35 GCF included BGCs for both the strawberry HST AF-toxin and pear HST AK-toxin, which were predicted in *Curvularia* (4/7) and section *Alternaria* (6/86) genomes. Previous work suggests that seven genes are included in the AK-toxin (AKT) gene cluster, responsible for the biosynthesis of AK-toxins I and II [15, 60]. The *AKT* backbone gene that synthesizes the EDA moiety has high similarity to *AFT* backbone gene producing the closely related AF-toxin [15, 60]. However, the MIBiG entry for AK-toxin I (BGC0001262) was incomplete, so BGC matches to compounds were inferred from more detailed examination of antiSMASH and BLASTp results as a whole, revealing important differences between *Curvularia, A. gaisen*, and other *Alternaria* species.

For *Alternaria* strains, the backbone PKS gene matched with 96.1-97.4% identity to the described AF-toxin PKS gene *AFT16-1* (NCBI V5Y0F7). However, since this backbone gene is shared between AF- and AK-toxin BGCs, we relied on accessory gene content to predict associated compounds. Two accessory genes unique to the *A. gaisen* genomes returned best hits to genes from the AK-toxin BGC: an enoyl-CoA hydratase with 100% identity to *AKT6-1* and a cytochrome P450 with 99.6% identity to *AKT7*. In addition, all three *A. gaisen* strains were isolated from a pear host, further suggesting these BGCs encode for the pear-specific AK-toxin. For the three non-*gaisen Alternaria* strains (*A. alternata*, *A. longipes*), a cytochrome P450 gene returned a best hit (94.0% identity) to *AFT11-1*, an accessory gene from the AF-toxin BGC. Although these results indicate these *Alternaria* strains may produce AF-toxin, all three of them were isolated from citrus, not strawberry hosts. For *Curvularia*, the sequence of the backbone PKS gene was, on average, only 78.5% identical to that of the *Alternaria* strains, indicating it was fairly divergent. Also, an acyl-CoA dehydrogenase accessory gene specific to *Curvularia* returned a best hit to *AFT10-1* from the AF-toxin BGC, but with only 85.6% identity. Taken together, it appears that *Curvularia* BGCs in PKSI 35 encode a compound that is closely related to, but distinct from, AF- or AK-toxin. Further genetic and chemical work would be needed to determine if the BGCs produce different compounds, or if the difference in *Curvularia* and *Alternaria* sequences is simply the result of taxa-specific divergence.

#### AM-toxin

The GCF for apple HST AM-toxin (NRPS 37), also called alternariolide, was found only in six apple-host isolates in section *Alternaria*. Although the AM-toxin MIBiG entry (MIBiG: BGC0001261) consists of only a backbone NRPS gene (*AMT1*), other genes such as *AMT2* (aldo–keto reductase), *AMT3* (cytochrome p450), and *AMT4* (thioesterase) are implicated in AM-toxin biosynthesis [61, 62]. The backbone genes from NRPS 37 matched with 97–99% identity to *AMT1*, a 13.1 kb gene essential for producing the cyclic peptide structure of AM-toxin [63]. BGCs from two *A. alternata* strains (FERA1166 and FERA1177) included an additional neighbouring cluster of nine genes. One of these additional genes was classified as “NRPS-like”, and was 99.2% identical to the *AMT10* gene. The exact role of this NRPS-like gene in AM-toxin biosynthesis is still unclear [61].

#### HC-toxin

Two genomes were predicted to have BGCs that produce HC-toxin (NRPS 63), a maize-targeting HST found originally in the plant pathogenic fungus *Helminthosporium carbonum* (synonyms *Cochliobolus carbonum*, *B. zeicola*). The *TOX2* locus controlling HC-toxin production extends over 540 kb and contains multiple copies of several genes [64]; however, the MIBiG entry for HC-toxin (BGC0001166) included just a single gene, the backbone NRPS gene *HTS1*.

For *B. zeicola* 26-R-13, the backbone NRPS was 99.9% identical to the reference *HTS1* gene from the same species. An immediately adjacent accessory gene was 79.8% identical to the HC-toxin major facilitator superfamily (MFS) transporter from *A. jesenskae*, and likely corresponds to the TOXA efflux pump described from *B. zeicola* [65]. For *A. brassicae* J3, there were two copies of the HC-toxin BGC, similar to that described for the only known HC-toxin producing strain of *Alternaria*, *A. jesenskae* [66]. The first copy had a backbone NRPS gene that was 97.8% identical to the *A. jesenskae* reference, with two cytochrome P450 genes and the TOXA MFS transporter homolog (91.7% identity). The second copy of the HC-toxin BGC had the backbone gene split into three segments, suggesting that the full reading frame may be disrupted, and that this copy may be undergoing degeneration after duplication (pseudogenization).

### Precautions for predicting compounds produced by BGCs

Detailed inspection of our results revealed that seemingly strong antiSMASH hits to known compounds could not always be validated. For instance, 11 of 15 BGCs in PKSI 27 were predicted to encode 6-methylsalicyclic acid (6-MSA) with 100% similarity, while three BGCs matched to patulin with 13% similarity and one BGC matched to pyranonigrin E with 100% similarity. These apparently incongruous results can be explained by the underlying meaning of the antiSMASH-based “percent similarity” score, which is calculated as the percentage of genes, from the most similar known cluster, that match genes in the query cluster. For example, the known BGC for patulin contains 15 genes (MIBiG accession: BGC0000120), and two of them matched genes in the query cluster, thus returning a 13% similarity value. However, the MIBiG entries for both 6-MSA (MIBiG accession: BGC0001276) and pyranonigrin E (MIBiG accession: BGC0001124) each contain only a single gene, which significantly matched one gene in the query BGCs. This 1/1 gene match resulted in similarity values of 100%, even though there are ~10 genes in the query cluster. Furthermore, the quality of the match between the query and reference genes must be thoroughly evaluated. Although 11 BGCs matched to 6-MSA with “100% similarity”, the single query gene matched to the database with only 58% sequence identity. However, the fact that these BGCs were clustered into the same GCF by BiG-SCAPE suggests they may be more similar than they appear from the antiSMASH results alone. 6-MSA is a simple polyketide produced by the 6-MSA synthase enzyme and a precursor to many SMs, including the patulin [67]. It is hypothesized that the 15-gene patulin BGC originated from a series of duplication and functional divergence events of 6-MSA-based BGCs, explaining why 6-MSA and patulin might be assigned to the same GCF [68, 69].

### Multiple GCFs corresponding to the same known compound

In contrast to the above example, some BGCs that returned the same “most similar known cluster” hit were grouped into separate GCFs by BiG-SCAPE. This situation was observed in five cases (Table 2.): curvupallide-B, dimethylcoprogen, equisetin, phomasetin, and solanapyrone D. In each case, no GCF members overlapped (i.e. the same BGC did not belong to multiple GCFs) and assignment to different GCFs often correlated with taxonomic groupings. Separation of related BGCs into distinct GCFs suggests significant differences in gene sequence, protein domain content, or protein domain synteny. A comparison of dimethylcoprogen (Fig. 4B) and phomasetin (Fig. 4C) BGCs revealed differences in gene cluster size, gene membership, orientation, and/or regions of homology, usually in the accessory genes. When multiple GCFs were linked to the same SM, it often included a mixture of NRPS and/or PKS-NRPS Hybrid classes, demonstrating how the detection of other nearby backbone genes can influence the automated classification of BGCs. For example, the dimethylcoprogen NRPS backbone could be located in close enough proximity to a type-1 PKS BGC to be considered together, as a PKS-NRPS Hybrid BGC (Fig. 4B). Such variation in overall BGC structure led to the calling of four different dimethylcoprogen GCFs, but each taxonomic sub-group of strains had the full complement of 100% prevalence when GCFs were summed. Singletons and other low-prevalence GCFs were sometimes separated from larger GCFs matching to the same compounds. For example, the single BGC in NRPS 169 from *Decorospora* had the phomasetin backbone gene (Fig. 4C), but was missing the majority of accessory genes, and was therefore distinct enough to be excluded from the main phomasetin GCF.

## Discussion

*Alternaria* species, and those in other genera in the family Pleosporaceae, are relevant to agriculture due to their high prevalence in the environment and ability to cause disease in economically important plants. These fungi can also produce SMs that cause food spoilage and compromise food safety. A major obstacle to establishing regulatory standards is the lack of a thorough assessment of the full range of SM production potential by *Alternaria* and close relatives. To date, systematic genome mining of fungal BGCs has mostly been applied to smaller datasets of restricted taxa that often draw conclusions from a single genome per species [69–73]. More recently, Gluck-Thaler et al. 2020 [74] analyzed 101 Dothideomycete taxa, one genome per species, which included only 12 Pleosporaceae species. Another study by Robey et al. 2021 [75] analyzed BGCs from 1000 fungal genomes but included only 23 Pleosporaceae genomes. Here we report the analyses of 187 Pleosporaceae genomes, making this the most comprehensive study of BGC identification, distribution, and diversity in this important fungal family. Our dataset also incorporates multiple genomes per species, revealing the high levels of BGC diversity between closely related strains at the intra-specific or population level.

The main goal of this research was to systematically characterize the nature and distribution of SM BGCs across the fungal genus *Alternaria*, which represented two-thirds of our genome sample. Thirty-seven novel *Alternaria* assemblies are being released here, significantly expanding the breadth and depth of genomic resources available for *Alternaria*. Increased sampling of *Alternaria* sections *Inf/Pse*, and the first analyzed genomes from sections *Embellisia, Ulocladium*, and *Pseudoulocladium*, provided valuable insight into these understudied sections of the genus. The remaining one-third of the dataset included taxa from seven other closely related genera in the family Pleosporaceae, allowing us to place the *Alternaria* BGC distribution results in phylogenetic and biological context. This study is essential for understanding and assessing the toxicological risk of *Alternaria*. These results can inform trade and food safety practices, and support the development of BGC-specific molecular diagnostic tools for broad-scale detection.

High-level analyses of GCF presence/absence (Fig. 5) allowed the visualization of the distribution of BGC features across all strains, at different scales of resolution. The patterns were complex, but in general, a good correlation between GCF distribution and phylogenetic relationship was observed throughout all levels of the taxonomic hierarchy. Groups of closely related taxa often displayed similar GCF content patterns. Some GCFs were well conserved within, and unique to, a small group of taxa, showing promise as potential chemotaxonomic identifiers (see below). Many GCFs had a patchy distribution across the phylogeny, demonstrating abundant diversity even amongst closely related strains and emphasizing the importance of including multiple genomes per species in the analyses. A high proportion of GCFs (54.0%) were unique to single genomes, indicating that we are not yet nearing the saturation point of BGC discovery in this group of fungi. Also, the vast majority of GCFs (94.9%) were uncharacterized and had no known predicted compounds, highlighting the need for expanded investigation of BGCs and their products.

The broadened sampling of *Alternaria* genomes in this dataset allowed for patterns to be studied with higher resolution, revealing distinct BGC profiles compared to other genera and between *Alternaria* sections. Among the 143 *Alternaria*-specific GCFs (Table 3), none were present across the entire genus (Fig. 6). Furthermore, none of the genus-specific GCFs present in sections *Inf*/*Pse* were found in other *Alternaria* sections, indicating that these sections likely have different and potentially diagnostic SM profiles. Unfortunately, the majority of *Alternaria*-specific GCFs did not match to named compounds. While sections *Inf/Pse* are phylogenetically and morphologically divergent from other *Alternaria* sections [18], they are closely related and quite similar to each other [10, 76, 77]. The clustering patterns of these sections in the GCF network diagrams (Figs. 2 and 3) and presence/absence heatmap (Fig. 5) match reports of their ability to produce a variety of SMs that are distinct from other *Alternaria* sections [19, 76, 78, 79]. Novae-zelandin A/B, 4Z-infectopyrone, and infectopyrone have been proposed as potential chemotaxonomic identifiers for sections *Inf/Pse* [79–81]. Structurally, all four compounds are α-pyrone derivatives and polyketides [20, 81–83], falling into the same category as ACRL-toxin, AOH, and other known biologically active metabolites. It is possible that novae-zelandin A/B, 4Z-infectopyrone, and infectopyrone may also have phytotoxic activity [20, 79, 84, 85]. The cognate BGCs for these SMs have yet to be identified; of the nine GCFs unique to sections *Inf/Pse*, seven are of the class PKSI or PKS-NRPS Hybrids, and therefore represent prime candidates for future investigation.

Five of the *Alternaria*-specific GCFs were conserved across the majority of *Alternaria* strains (52.8—76.4% prevalence, Table 3). While none reached 100% prevalence within *Alternaria* sub-groups either, some showed differences between sub-groups (e.g. unique to section *Alternaria*). Such distribution patterns, across the genus or across sections, suggest vertical inheritance, in which the BGC was present in a recent common ancestor and was propagated to the extant lineages, with sporadic losses in some strains [68, 73, 74].

One GCF of particular interest was PKSI 5, which corresponds to AOH, the main *Alternaria* toxin of concern regarding food safety [22]. The BGC responsible for the production of AOH also produces AME, a related mycotoxin commonly found in foods [52]. Whereas previous studies on AOH were limited to a handful of strains from section *Alternaria* [51, 52, 86], we investigated the distribution of the AOH BGCs in 123 *Alternaria* strains, spanning at least 28 species across nine sections. The AOH/AME BGC was restricted to strains in sections *Alternaria* and *Porri*, and the monotypic lineage *A. brassicae*, and was not found in any other *Alternaria* sections or genera included in this analysis. Accurate knowledge of the limited distribution the AOH BGC significantly narrows down the scope of species to screen for when implementing monitoring and handling measures for *Alternaria* foodborne toxins. Despite their significance, there are currently no guidelines or legislative restrictions anywhere in the world on the allowable amount of *Alternaria* toxins in food or feed. Only the European Union has prescribed a threshold of toxicological concern of 1500 ng/kg body weight (bw) per day for tenuazonic acid and tentoxin, and only 2.5 ng/kg bw per day for AOH and AME. However, recent estimates place exposure ranges between 3.8—71.6 ng/kg bw per day AOH, and 3.4—38.8 ng/kg bw per day for AME [22], mainly from fruits and vegetables which are commonly susceptible to *Alternaria* infection. Furthermore, these toxins may remain in derived food products because AOH and AME do not deteriorate under many standard food processing conditions [87, 88]. While there is certainly a need for more compound-specific toxicity data to better understand the risk to consumers, provisional monitoring of sections *Porri* and *Alternaria* species appears to be justified based on the evidence to date.

From a phytosanitary perspective, the Canadian Food Inspection Agency regulates only two species of *Alternaria*: *A. gaisen* and *A. kikuchiana* (= *A. gaisen*), originally thought to be the causal agents of black spot of Asian pear [14, 39, 89]. However, it has been demonstrated that the production of AK-toxin is, in fact, responsible for the infection of susceptible pear varieties, rather than the particular species producing it [11, 14, 90, 91]. Related work has shown that ACT-toxin II, the citrus HST, is also highly toxic to susceptible pear varieties [13, 59]. This cross-host pathogenicity can be explained by the structural similarity of the AK- and ACT-toxins; at least three other genes involved in the production of ACT-toxin II have homologous genes in the AK-toxin BGC. The AK-toxin BGC was present in three of the four *A. gaisen* genomes examined here, all of which were isolated from pear hosts. While this study would benefit from a larger sample size of *A. gaisen* and other underrepresented *Alternaria* species, these preliminary findings suggest that AK-toxin is restricted to *A. gaisen* and reinforces the status of the species as a phytosanitary pest by the CFIA. Unlike the blanket regulation of *A. gaisen*, certain strains of *A. alternata* and *A. longipes* may also pose a risk to Asian pear and should be monitored in detail with molecular testing.

BGCs with a highly irregular distribution throughout *Alternaria* or other genera, as is commonly observed with HST BGCs, could be attributed to horizontal gene transfer. HST BGCs often reside on conditionally dispensable chromosomes (CDCs) that can be inherited horizontally and/or lost, causing strains to become pathogenic or non-pathogenic to target hosts [11, 61, 92]. The presence of supposed *Alternaria*-specific HSTs in other Pleosporaceae genera further demonstrates that pathotype-conferring genetic components may not always correlate with taxonomic identity (i.e. pathotypes can be polyphyletic). Friesen et al. 2008 [93] hypothesized that species in the order Pleosporales, predominantly composed of fungal plant pathogens, are able to share HST genes by lateral transfer, even between different genera. Indeed, recent studies have reported clear evidence for lateral transfer among well-studied fungal pathogens beyond Pleosporales [73, 94, 95]. As HSTs are linked with increased virulence, it remains a priority to determine the SM profiles and pathogenicity of such *Alternaria* species [11, 58, 73]. Further research on CDC sequences could reveal diagnostic DNA signatures, translating into the development of molecular detection tools and mitigation of phytosanitary risk from *Alternaria*.

Gapless, chromosome-level genome assemblies would reveal the genomic location of BGCs of interest (i.e. on main chromosomes or CDCs), allowing higher-confidence predictions for horizontal gene transfer. For example, an HST BGC on a small CDC is more likely to be gained or lost, when compared to a BGC embedded centrally in a core chromosome. One obvious limitation of our study is that conclusions are based on the assumption that the failure to detect a BGC represents true absence. We acknowledge that de novo assembly of short-read sequence data may result in incomplete assemblies (e.g. due to highly repetitive regions), which can impair the accuracy for full BGC identification, particularly near contig ends. Furthermore, if a single BGC is broken apart across multiple contigs, this could lead to the artificial inflation of numbers of detected BGCs and/or GCFs. While we did restrict our analyses to apparently high-quality genomes, we cannot exclude the possibility that the absence of a GCF was a technical artefact caused by incomplete genome assemblies.

In this study, we applied commonly employed tools for BGC detection, annotation, and functional characterization to our Pleosporaceae dataset. We also presented several cautionary examples demonstrating how the results of these methods cannot always be taken at face value, and thorough examination of additional relevant evidence is often required before firm conclusions can be drawn. For example, an antiSMASH-based “percent similarity” score of 100% does not necessarily mean the associated compound is produced by that BGC. This “percent similarity” is calculated as the percentage of genes, from the most similar known cluster, that match genes in the query cluster. Therefore, known BGCs with relatively simple gene structures (e.g. only one gene) are much more likely to return 100% similarity hits, when compared to highly complex known BGCs with several included genes. Furthermore, antiSMASH returns the closest match given the information currently available in databases. Similar to BLAST-searching of sequence databases, understanding the limitations of what can be inferred from “best hits” is important to the interpretation of the results. Another point of concern was how different methods of clustering BGCs into GCFs could give quite different results. We had to test various algorithms and clustering parameters using BiG-SCAPE, AutoAnnotate, and Cytoscape, to determine the optimal settings that gave reliable and biologically meaningful results. For example, when multiple BGCs linked to the same compound are clustered into different GCFs, their separation should align with logical and informative factors such as taxonomy, accessory gene content, and sequence similarity.

## Conclusions

Our genome mining study presents a broad summary of the distribution of BGCs, GCFs, and associated compounds in the family Pleosporaceae, but also generates multiple new questions regarding the secondary metabolism of *Alternaria* and closely related taxa. For instance, why does *Setosphaeria* have so many PKS BGCs, or why does *Pyrenophora* have such a variable number of NRPS BGCs? What is the functional significance of 93% of the RiPP BGCs being found in *Alternaria*? There are clear subgeneric SM BGC profiles in *Alternaria* and *Pyrenophora*—what classes of BGCs are the most different, and what important compounds do they produce? Do taxon-specific BGCs and associated compounds have high enough prevalence to function as reliable chemotaxonomic markers? The results of SM BGC genome mining presented here can act as a launching point for further targeted research into the toxicological potential of these fungi. There are at least 27 sub-generic sections in *Alternaria*, but only nine sections (and one monotypic lineage) were included in these analyses, some of which were represented by only one strain. Similarly, the sample sizes for some other genera like *Decorospora, Paradendryphiella,* and *Setosphaeria* were limited. Future work would benefit from increased genome sampling of other *Alternaria* sections and related genera to reach saturation of SM BGC diversity. As more Pleosporaceae whole genomes become available, they can be incorporated into these analyses to expand and revise BGC profiles, potentially reinforcing the distributional patterns observed here. There are over 70 *Alternaria* SMs with identified chemical structures and bioactivities [20]; however, many of these compounds have not yet been matched to a gene cluster. Likewise, genome mining efforts may identify thousands of SM BGCs, but only a small proportion of them can be matched to a putative compound based on the existing reference databases. Profiling the production of SMs using mass spectrometry and other metabolomics tools is another perspective from which the results of this work could be investigated. Integrative analyses of such high-resolution, paired genomic and metabolomic datasets could vastly improve our ability to make links between BGCs and specific compounds, thereby increasing the accuracy and overall value of these types of studies.

## Supplementary Information

Supplementary Material 1

## Data Availability

The datasets generated and/or analyzed during the current study are available in the NCBI and JGI databases under the accession numbers listed in Table S1.
