# Supplementary material for: Genome mining reveals the distribution of biosynthetic gene clusters in Alternaria and related fungal taxa within the family Pleosporaceae
Source: BMC Genomics. 2025 Jul 21;26:678. doi: 10.1186/s12864-025-11754-z (PMC12278603; doi:10.1186/s12864-025-11754-z)
Supplement: Supplementary file 1 — Supplementary Material 1 [file 12864_2025_11754_MOESM1_ESM.pdf]

SUPPLEMENTARY MATERIAL

Genome Mining Reveals the Distribution of Biosynthetic Gene Clusters in Alternaria and Related Fungal Taxa within the Family Pleosporaceae

by Natalie E. Kim and Jeremy R. Dettman

Contents:

Fig. S1 – GCF networks for terpenes, PKSOther, and RiPPs classes.
Table S1 - Assembly and annotation statistics for all 187 Pleosporaceae genomes.
Table S2 - List of programs and versions used in this study.
Table S3 - Summary of gene prediction results from optimization trials.
Table S4 – Numbers of genomes included in analyses, by taxonomic group.
Table S5 - Composition of higher-level BGC classes.
Table S6 - Prevalence of all 548 GCFs across 187 taxa divided into four groups.
Supplementary Text
Supplementary References

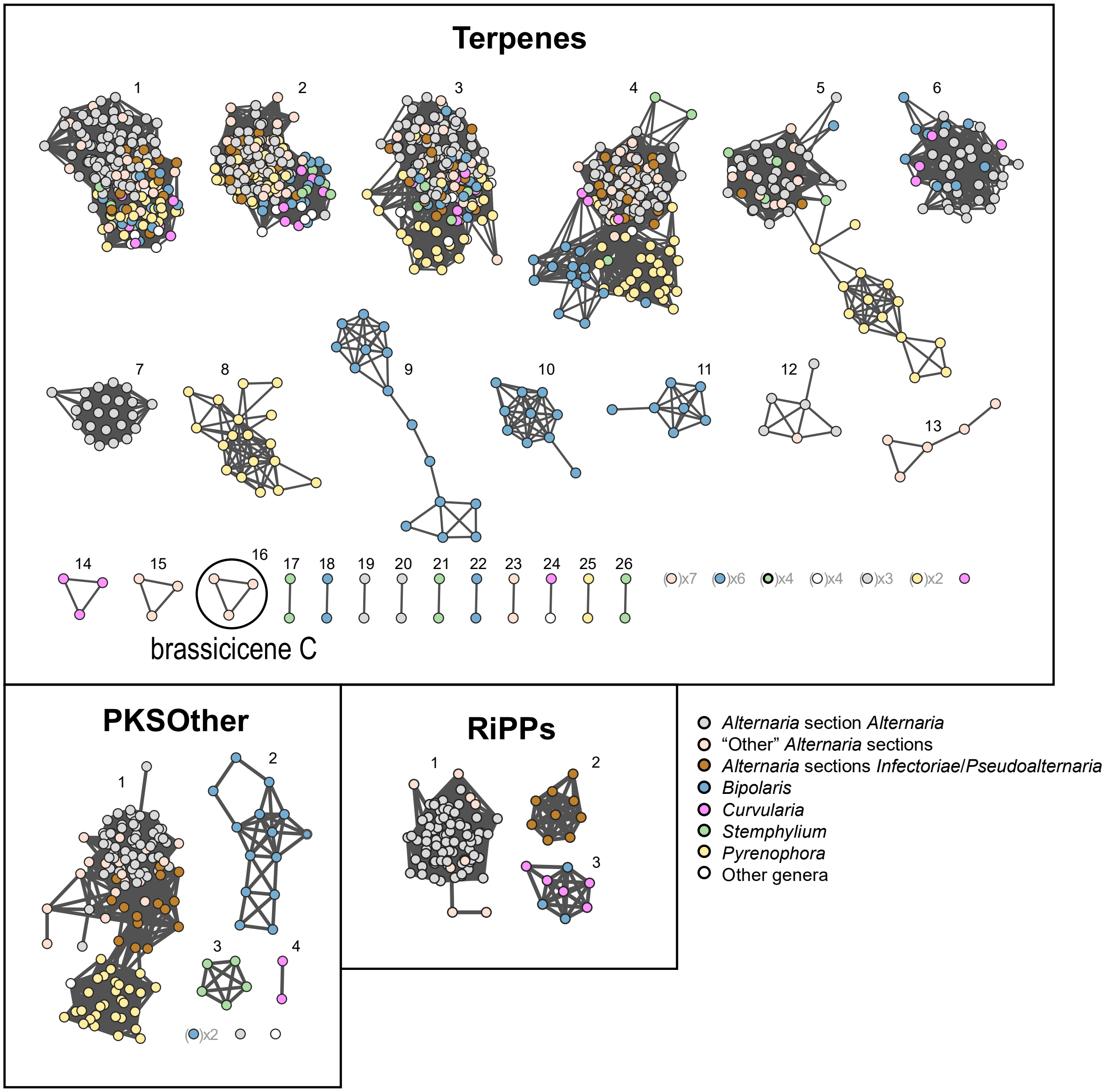

Fig. S1 Gene cluster family (GCF) networks for the terpenes, PKSOther, and RiPPs classes. GCFs are numbered according to the named GCFs and displayed in descending order of network size. Each node represents a BGC and lines (edges) connect nodes with distances less than or equal to the cutoff threshold (0.4). Nodes are color-coded by taxonomic group, as shown in the legend. Singletons are displayed as a representative single node, with the number of such singletons indicated beside each single node. GCFs associated with known compounds are circled and annotated.

Table S1 Assembly and annotation statistics for all 187 Pleosporaceae genomes used in this study.

Species	Alternaria Section	Strain	Accession	Source	Assembly length (Mb)	Contigs (#)	N50 (kb)	Gene Count	BGC count	BUSCO completeness (%)	GC%	TE content (%)
Alternaria alternata	*Alternaria*	ATCC11680	Alalt1	JGI	33.52	253	451	12,250	31	99.0	51.1	1.6
Alternaria alternata	*Alternaria*	ATCC34957	GCA_001443195.2	NCBI	33.50	27	2,828	12,078	27	98.7	51.0	2.1
Alternaria alternata	*Alternaria*	ATCC66891	Alalte1	JGI	33.15	278	304	12,093	26	98.9	51.2	1.7
Alternaria alternata	*Alternaria*	B2a	GCA_001696825.1	NCBI	33.01	530	244	11,981	27	97.7	51.3	1.5
Alternaria alternata	*Alternaria*	B3	GCA_014154925.1	NCBI	33.84	76	1,479	12,210	26	99.1	51.0	2.5
Alternaria alternata	*Alternaria*	BMP0270	Aalte1	JGI	33.40	171	757	12,083	27	99.2	51.0	1.9
Alternaria alternata	***Alternaria***	**DET2001**	**JAAOQX010000000**	**DET**	**33.47**	**111**	**1,206**	**12,151**	**28**	**99.3**	**51.0**	**2.1**
Alternaria alternata	***Alternaria***	**DET2010**	**JAAOQV010000000**	**DET**	**33.79**	**216**	**1,367**	**12,219**	**29**	**99.2**	**51.0**	**2.3**
Alternaria alternata	***Alternaria***	**DET2019**	**JAAOQU010000000**	**DET**	**35.17**	**449**	**824**	**12,565**	**30**	**99.1**	**51.0**	**2.7**
Alternaria alternata	***Alternaria***	**DET2071**	**JAERPG000000000**	**DET**	**33.43**	**60**	**2,419**	**12,130**	**30**	**99.1**	**51.1**	**2.0**
Alternaria alternata	***Alternaria***	**DET2076**	**JAERPE000000000**	**DET**	**33.81**	**144**	**2,644**	**12,244**	**29**	**99.0**	**51.0**	**2.1**
Alternaria alternata	***Alternaria***	**DET2080**	**JAERPC000000000**	**DET**	**33.50**	**63**	**1,920**	**12,110**	**28**	**99.2**	**51.0**	**2.1**
Alternaria alternata	***Alternaria***	**DET2092**	**JAERPA000000000**	**DET**	**33.37**	**77**	**1,557**	**12,128**	**27**	**99.4**	**51.0**	**2.1**
Alternaria alternata	***Alternaria***	**DET2110**	**JAEROT000000000**	**DET**	**33.89**	**137**	**2,213**	**12,318**	**28**	**99.2**	**51.1**	**2.0**
Alternaria alternata	***Alternaria***	**DET2123**	**JAEROQ000000000**	**DET**	**33.82**	**168**	**1,281**	**12,246**	**28**	**99.4**	**51.1**	**2.3**
Alternaria alternata	*Alternaria*	FERA1177	GCA_004154755.1	NCBI	35.63	735	537	12,675	30	99.0	51.1	2.8
Alternaria alternata	*Alternaria*	JS-0527	GCA_011420255.1	NCBI	33.80	18	3,059	12,356	27	96.1	50.9	3.0
Alternaria alternata	*Alternaria*	JS-1623	GCA_009650635.1	NCBI	33.67	11	4,962	12,448	24	91.2	51.0	2.4
Alternaria alternata	***Alternaria***	**KAS5299**	**JAEROD000000000**	**DET**	**34.14**	**147**	**2,083**	**12,321**	**28**	**99.2**	**51.1**	**2.5**
Alternaria alternata	***Alternaria***	**KAS5303**	**JAEROC000000000**	**DET**	**33.72**	**88**	**2,569**	**12,232**	**28**	**99.2**	**51.1**	**2.1**
Alternaria alternata	***Alternaria***	**KAS5306**	**JAEROB000000000**	**DET**	**34.03**	**174**	**2,058**	**12,217**	**28**	**99.2**	**51.1**	**2.4**
Alternaria alternata	***Alternaria***	**KAS5313**	**JAEROA000000000**	**DET**	**33.68**	**112**	**1,298**	**12,153**	**27**	**99.4**	**51.0**	**2.4**
Alternaria alternata	***Alternaria***	**KAS5320**	**JAERNZ000000000**	**DET**	**33.46**	**80**	**1,536**	**12,122**	**28**	**99.3**	**51.0**	**2.0**
Alternaria alternata	***Alternaria***	**KAS5372**	**JAERNW000000000**	**DET**	**33.57**	**173**	**1,006**	**12,148**	**27**	**99.3**	**51.1**	**1.8**
Alternaria alternata	***Alternaria***	**KAS5386**	**JAERNV000000000**	**DET**	**34.23**	**129**	**1,784**	**12,327**	**32**	**99.3**	**51.0**	**2.5**
Alternaria alternata	***Alternaria***	**KAS5394**	**JAERNU000000000**	**DET**	**33.50**	**110**	**2,121**	**12,106**	**29**	**99.2**	**51.0**	**2.1**
Alternaria alternata	***Alternaria***	**KAS5428**	**JAERNR000000000**	**DET**	**33.34**	**52**	**1,649**	**12,082**	**26**	**99.3**	**51.0**	**2.1**
Alternaria alternata	***Alternaria***	**KAS5468**	**JAERNO000000000**	**DET**	**34.46**	**272**	**1,639**	**12,456**	**30**	**99.0**	**51.1**	**3.0**
Alternaria alternata	***Alternaria***	**KAS5489**	**JAERNJ000000000**	**DET**	**34.21**	**224**	**2,440**	**12,329**	**28**	**99.1**	**51.1**	**2.8**
Alternaria alternata	***Alternaria***	**KAS5497**	**JAERNI000000000**	**DET**	**34.02**	**250**	**1,556**	**12,275**	**29**	**99.3**	**51.0**	**2.5**
Alternaria alternata	***Alternaria***	**KAS5499**	**JAERNH000000000**	**DET**	**34.07**	**177**	**1,644**	**12,314**	**26**	**99.3**	**51.1**	**2.7**

Alternaria alternata	***Alternaria***	**KAS5513**	**JAERNF000000000**	**DET**	**33.80**	**252**	**1,066**	**12,224**	**27**	**99.2**	**51.1**	**2.7**
Alternaria alternata	***Alternaria***	**KAS5516**	**JAAOQT010000000**	**DET**	**33.95**	**174**	**1,478**	**12,251**	**26**	**99.2**	**51.0**	**2.4**
Alternaria alternata	***Alternaria***	**KAS5743**	**JAERND000000000**	**DET**	**34.11**	**211**	**1,355**	**12,306**	**30**	**99.1**	**51.1**	**2.6**
Alternaria alternata	*Alternaria*	MOD1-FUNGI5	GCA_004634295.1	NCBI	33.40	1,085	71	12,188	30	97.8	51.3	1.4
Alternaria alternata	*Alternaria*	MPI-PUGE-AT-0064	Altalt1	JGI	33.44	26	3,089	12,075	27	99.0	51.1	2.1
Alternaria alternata	*Alternaria*	NAP07	GCA_009932595.1	NCBI	35.84	19	3,071	12,604	35	99.1	51.0	5.2
Alternaria alternata	*Alternaria*	PF1	GCA_018104255.1	NCBI	34.86	15	3,162	12,473	29	98.9	51.0	3.0
Alternaria alternata	*Alternaria*	PN1	GCA_011420445.1	NCBI	33.77	14	3,094	12,248	24	93.7	51.0	2.8
Alternaria alternata	*Alternaria*	PN2	GCA_011420565.1	NCBI	33.53	15	3,099	12,177	27	90.8	51.0	2.9
Alternaria alternata	*Alternaria*	SRC1lrK2f	GCA_001642055.1	NCBI	32.99	79	1,098	12,193	28	99.4	51.4	1.2
Alternaria alternata	*Alternaria*	Z7	GCA_014751505.1	NCBI	34.28	12	3,085	12,241	29	99.1	51.0	2.6
Alternaria alternata (Alternaria citriarbusti)	*Alternaria*	BMP2343	Altci1	JGI	33.85	1,269	48	12,282	32	96.9	51.0	2.1
Alternaria alternata (Alternaria limoniasperae)	*Alternaria*	BMP2335	Altli1	JGI	34.76	1,596	50	12,492	31	96.9	51.0	2.5
Alternaria alternata (Alternaria mali)	*Alternaria*	BMP3064	Amal3064	JGI	34.33	1,669	36	12,413	33	94.4	50.8	2.2
Alternaria alternata (Alternaria tenuissima)	*Alternaria*	ANJ	GCA_017589455.1	NCBI	33.68	194	1,434	12,167	27	99.3	51.1	2.0
Alternaria alternata (Alternaria tenuissima)	*Alternaria*	BMP0304	Altte1	JGI	33.36	216	662	12,124	28	99.2	51.0	1.8
Alternaria alternata (Alternaria tenuissima)	*Alternaria*	FERA1082	GCA_004154745.1	NCBI	33.94	345	547	12,216	29	99.0	51.1	2.3
Alternaria alternata (Alternaria tenuissima)	*Alternaria*	FERA1164	GCA_004156015.1	NCBI	34.72	250	709	12,301	29	99.3	50.9	3.0
Alternaria alternata (Alternaria tenuissima)	*Alternaria*	FERA1166	GCA_004156035.1	NCBI	35.70	22	2,584	12,586	34	99.0	51.1	4.3
Alternaria alternata (Alternaria tenuissima)	*Alternaria*	FERA24350	GCA_004154735.1	NCBI	33.07	167	608	12,114	27	99.2	51.3	1.5
Alternaria alternata (Alternaria tenuissima)	*Alternaria*	FERA635	GCA_004168565.1	NCBI	36.06	912	319	12,734	37	99.2	50.9	3.5
Alternaria alternata (Alternaria tenuissima)	*Alternaria*	FERA648	GCA_004154765.1	NCBI	33.51	124	1,341	12,058	29	99.3	51.0	2.3
Alternaria alternata (Alternaria tenuissima)	*Alternaria*	FERA743	GCA_004154845.1	NCBI	35.92	788	622	12,683	35	99.2	51.1	3.0
Alternaria arborescens species complex	*Alternaria*	BMP0308	Altar1	JGI	33.02	6,397	8	13,505	33	80.2	51.1	2.1

***Alternaria arborescens* species complex**	***Alternaria***	**DET2008**	**JAAOQW010000000**	**DET**	**33.30**	**127**	**817**	**11,993**	**30**	**99.1**	**51.0**	**2.0**
***Alternaria arborescens* species complex**	***Alternaria***	**DET2035**	**JAERPI000000000**	**DET**	**33.10**	**163**	**672**	**11,982**	**30**	**99.3**	**51.1**	**1.8**
***Alternaria arborescens* species complex**	***Alternaria***	**DET2078**	**JAERPD000000000**	**DET**	**33.94**	**292**	**1,364**	**12,138**	**32**	**99.2**	**50.9**	**2.5**
***Alternaria arborescens* species complex**	***Alternaria***	**DET2085**	**JAERPB000000000**	**DET**	**33.57**	**213**	**1,051**	**12,096**	**30**	**99.2**	**51.0**	**2.0**
***Alternaria arborescens* species complex**	***Alternaria***	**DET2119**	**JAEROR000000000**	**DET**	**33.60**	**155**	**1,886**	**12,097**	**31**	**99.1**	**51.0**	**2.1**
Alternaria arborescens species complex	*Alternaria*	EGS39-128	GCA_000256225.1	NCBI	33.79	493	314	12,051	31	99.1	50.9	2.7
Alternaria arborescens species complex	*Alternaria*	FERA675	GCA_004154835.1	NCBI	33.94	325	863	12,117	27	99.1	51.1	2.7
***Alternaria arborescens* species complex**	***Alternaria***	**KAS5275**	**JAEROF000000000**	**DET**	**34.25**	**236**	**1,355**	**12,231**	**34**	**99.2**	**50.9**	**2.9**
***Alternaria arborescens* species complex**	***Alternaria***	**KAS5321**	**JAERNY000000000**	**DET**	**33.00**	**125**	**667**	**11,956**	**31**	**99.3**	**51.1**	**1.9**
***Alternaria arborescens* species complex**	***Alternaria***	**KAS5368**	**JAERNX000000000**	**DET**	**33.69**	**216**	**1,054**	**12,072**	**30**	**99.2**	**51.0**	**2.5**
***Alternaria arborescens* species complex**	***Alternaria***	**KAS5398**	**JAERNT000000000**	**DET**	**33.95**	**196**	**987**	**12,029**	**27**	**99.2**	**50.9**	**3.3**
***Alternaria arborescens* species complex**	***Alternaria***	**KAS5399**	**JAERNS000000000**	**DET**	**34.16**	**215**	**1,140**	**12,153**	**29**	**99.2**	**50.9**	**3.1**
***Alternaria arborescens* species complex**	***Alternaria***	**KAS5521**	**JAERNE000000000**	**DET**	**33.67**	**229**	**1,241**	**12,073**	**31**	**99.1**	**51.0**	**2.5**
***Alternaria arborescens* species complex**	***Alternaria***	**KAS5762**	**JAERNC000000000**	**DET**	**33.58**	**211**	**1,041**	**12,048**	**30**	**99.1**	**50.9**	**2.4**
Alternaria arborescens species complex	*Alternaria*	MOD1-FUNGI6	GCA_004634205.1	NCBI	33.75	459	302	12,016	30	98.8	51.1	2.3
Alternaria arborescens species complex	*Alternaria*	NRRL20593	GCA_013282825.1	NCBI	33.59	701	134	12,087	31	97.9	51.2	2.2
Alternaria arborescens species complex	*Alternaria*	RGR97.0013	GCA_004155955.1	NCBI	33.80	287	685	12,005	27	99.1	50.9	2.9
Alternaria arborescens species complex	*Alternaria*	RGR97.0016	GCA_004154815.1	NCBI	33.77	339	528	12,025	29	99.1	51.1	2.8
Alternaria burnsii	*Alternaria*	CBS107.38	GCA_013036055.1	NCBI	32.95	53	1,806	11,833	26	99.1	50.9	1.5
Alternaria gaisen	*Alternaria*	BMP2338	Altga1	JGI	33.94	5,455	10	13,288	32	82.4	50.7	2.0
Alternaria gaisen	***Alternaria***	**EGS90-391**	**EGS90-391 (see Woudenberg et al. 2015)**	**DET**	**35.15**	**270**	**529**	**12,450**	**32**	**99.1**	**50.9**	**2.6**
Alternaria gaisen	*Alternaria*	FERA650	GCA_004156025.2	NCBI	34.35	27	2,110	12,224	29	99.1	51.1	2.9

Alternaria gaisen (Alternaria fragaria)	*Alternaria*	BMP3062	Altfr1	JGI	33.13	779	78	12,048	29	97.8	51.0	1.5
Alternaria gaisen (Alternaria mali)	*Alternaria*	BMP3063	Amal3063	JGI	33.61	2,697	21	12,371	31	90.8	50.8	2.1
Alternaria longipes	*Alternaria*	BMP0313	Altlo1	JGI	35.68	1,079	139	12,648	27	99.2	51.0	2.8
Alternaria longipes	*Alternaria*	CBS540.94	GCA_019059555.1	NCBI	39.43	15	3,599	13,055	34	99.0	51.0	10.4
Alternaria longipes	***Alternaria***	**KAS1274**	**JAEROG000000000**	**DET**	**33.76**	**79**	**1,282**	**12,054**	**26**	**99.3**	**50.9**	**2.2**
Alternaria longipes (Alternaria alternata)	*Alternaria*	EV-MIL-31	GCA_016097525.1	NCBI	34.96	61	2,282	12,386	34	99.0	51.0	2.5
Alternaria longipes (Alternaria tangelonis)	*Alternaria*	BMP2327	Altta1	JGI	33.75	1,639	37	12,133	30	94.8	50.9	1.9
Alternaria sectAlternaria*	***Alternaria***	**KAS6096**	**JAERMY000000000**	**DET**	**35.57**	**353**	**389**	**12,266**	**26**	**99.1**	**50.1**	**6.6**
Alternaria sectAlternaria*	***Alternaria***	**KAS6097**	**JAERMX000000000**	**DET**	**35.77**	**234**	**566**	**12,193**	**26**	**99.2**	**50.0**	**7.2**
Alternaria brassicicola	*Brassicicola*	Abra43	GCA_002796735.1	NCBI	31.04	29	2,102	9,816	26	98.9	50.9	9.4
Alternaria brassicicola	*Brassicicola*	Altbr1	Altbr1	JGI	31.97	816	2,486	9,568	26	92.8	50.5	8.6
Alternaria brassicicola	*Brassicicola*	ATCC96836	GCA_000174375.1	NCBI	29.54	3,276	19	10,107	24	87.1	50.7	8.4
Alternaria embellisia	***Embellisia***	**KAS5799**	**JAERNB000000000**	**DET**	**44.57**	**1,403**	**139**	**11,950**	**21**	**99.1**	**48.0**	**25.9**
Alternaria sectInfectoriae*	***Infectoriae***	**DET2042**	**JAERPH000000000**	**DET**	**36.50**	**114**	**1,642**	**12,510**	**27**	**99.3**	**50.2**	**7.8**
Alternaria sectInfectoriae*	***Infectoriae***	**DET2072**	**JAERPF000000000**	**DET**	**36.65**	**113**	**2,070**	**12,488**	**25**	**99.2**	**50.2**	**7.4**
Alternaria sectInfectoriae*	***Infectoriae***	**DET2105**	**JAEROX000000000**	**DET**	**33.68**	**47**	**2,370**	**12,454**	**28**	**99.2**	**52.3**	**2.0**
Alternaria sectInfectoriae*	***Infectoriae***	**DET2106**	**JAEROW000000000**	**DET**	**33.79**	**46**	**2,420**	**12,489**	**28**	**99.2**	**52.3**	**2.0**
Alternaria sectInfectoriae*	***Infectoriae***	**KAS5446**	**JAERNQ000000000**	**DET**	**33.48**	**285**	**1,053**	**12,437**	**27**	**99.1**	**52.4**	**1.7**
Alternaria sectInfectoriae*	***Infectoriae***	**KAS5449**	**JAERNP000000000**	**DET**	**33.77**	**41**	**2,355**	**12,418**	**27**	**99.2**	**52.2**	**1.8**
Alternaria sectInfectoriae*	***Infectoriae***	**KAS5470**	**JAERNN000000000**	**DET**	**33.75**	**55**	**1,888**	**12,444**	**27**	**99.3**	**52.2**	**2.5**
Alternaria sectInfectoriae*	***Infectoriae***	**KAS5477**	**JAERNL000000000**	**DET**	**33.62**	**31**	**2,373**	**12,459**	**27**	**99.2**	**52.3**	**1.8**
Alternaria sectInfectoriae*	***Infectoriae***	**KAS5486**	**JAERNK000000000**	**DET**	**33.71**	**89**	**2,050**	**12,454**	**27**	**99.2**	**52.2**	**2.2**
Alternaria sectInfectoriae*	***Infectoriae***	**KAS5506**	**JAERNG000000000**	**DET**	**33.72**	**120**	**1,998**	**12,461**	**26**	**99.2**	**52.4**	**2.0**
Alternaria brassicae	monotypic lineage	J3	GCA_004936725.1	NCBI	34.14	17	2,988	10,946	32	97.8	50.7	10.0
Alternaria capsici	*Porri*	BMP0180	Altca1	JGI	31.34	1,845	33	11,113	27	98.5	52.0	2.0

Alternaria carthami	*Porri*	BMP1963	Altcar1	JGI	32.67	1,241	77	11,150	35	96.8	52.0	2.1
Alternaria crassa	*Porri*	BMP0172	Altcr1	JGI	32.61	1,557	59	10,929	30	97.5	51.7	3.2
Alternaria dauci	*Porri*	BMP0167	Altda1	JGI	30.41	3,645	13	11,459	36	81.0	51.7	1.4
Alternaria macrospora	*Porri*	BMP1949	Altma1	JGI	31.34	1,649	37	11,040	34	94.4	52.3	1.3
Alternaria porri	*Porri*	BMP0178	Altpo1	JGI	29.19	5,082	9	10,598	31	68.3	52.0	1.1
Alternaria solani	*Porri*	BMP0185	Altso1	JGI	31.12	2,922	18	12,079	36	90.5	52.6	1.4
Alternaria solani	*Porri*	HWC-168-2012p	GCA_002837235.1	NCBI	32.83	58	2,613	11,364	32	99.0	51.2	4.8
Alternaria solani	*Porri*	NL03003	GCA_002952155.1	NCBI	32.78	10	2,867	11,389	30	99.1	51.3	5.3
Alternaria tomatophila	*Porri*	BMP2032	Alttom1	JGI	32.29	2,639	23	12,045	40	94.3	52.3	1.6
Alternaria rosae	*Pseudoalternaria*	MPI-PUGE-AT-0040	Altro1	JGI	33.83	52	3,278	11,980	28	99.0	52.0	4.8
Alternaria sectPseudoalternaria*	***Pseudoalternaria***	**DET2103**	**JAEROZ000000000**	**DET**	**32.97**	**87**	**1,641**	**11,970**	**26**	**99.1**	**52.2**	**2.1**
Alternaria sectPseudoalternaria*	***Pseudoalternaria***	**DET2104**	**JAEROY000000000**	**DET**	**32.78**	**96**	**1,314**	**11,871**	**27**	**99.3**	**52.1**	**2.3**
Alternaria sectPseudoalternaria*	***Pseudoalternaria***	**DET2107**	**JAEROV000000000**	**DET**	**32.76**	**102**	**1,459**	**11,887**	**27**	**99.3**	**52.1**	**2.3**
Alternaria sectPseudoalternaria*	***Pseudoalternaria***	**KAS5474**	**JAERNM000000000**	**DET**	**32.53**	**88**	**860**	**11,823**	**27**	**99.2**	**52.2**	**2.3**
Alternaria chartarum	***Pseudoulocladium***	**KAS5825-2**	**JAERNA000000000**	**DET**	**38.71**	**1,108**	**259**	**13,113**	**26**	**99.2**	**50.7**	**9.3**
Alternaria atra	*Ulocladioides*	CS162	GCA_907166805.1	NCBI	39.61	43	3,893	12,821	33	98.6	50.9	9.0
Alternaria atra	***Ulocladioides***	**KAS5298**	**JAEROE000000000**	**DET**	**35.62**	**425**	**395**	**11,795**	**30**	**99.0**	**50.6**	**7.3**
Alternaria atra	*Ulocladioides*	MOD1-FUNGI7	GCA_004634305.1	NCBI	34.82	927	163	11,795	32	98.6	50.9	5.2
Alternaria consortialis	*Ulocladioides*	JCM1940	GCA_001950455.1	NCBI	34.24	23	2,770	11,468	28	98.9	50.7	5.0
Alternaria cucurbitae	***Ulocladioides***	**KAS5913**	**JAERMZ000000000**	**DET**	**34.00**	**493**	**464**	**11,698**	**30**	**99.2**	**51.0**	**3.5**
Alternaria alternariae	***Ulocladium***	**KAS5756**	**JAEVYP000000000**	**DET**	**40.50**	**1,387**	**323**	**13,055**	**21**	**99.3**	**50.1**	**13.7**
Bipolaris cookei	n/a	LSLP18.3	GCA_002286855.1	NCBI	36.17	320	379	10,144	36	99.0	50.0	16.6
Bipolaris maydis	n/a	ATCC48331	GCA_000354255.1	NCBI	32.93	207	964	10,468	51	98.9	50.7	1.9
Bipolaris maydis	n/a	BM1	GCA_019454015.1	NCBI	36.23	27	1,859	10,515	47	98.7	49.1	14.2
Bipolaris maydis	n/a	C5	GCA_000338975.1	NCBI	36.46	68	1,842	10,857	50	98.7	49.8	10.8
Bipolaris maydis (Cochliobolus heterostrophus)	n/a	C4-1	CocheC4-1	JGI	32.93	207	964	10,500	51	99.0	50.7	2.0
Bipolaris maydis (Cochliobolus heterostrophus)	n/a	Hm540-1	ChetHm540-1	JGI	32.12	1,097	86	10,319	50	98.7	50.5	2.8

Bipolaris oryzae	n/a	ATCC44560	GCA_000523455.1	NCBI	31.36	619	134	10,145	38	98.8	50.5	3.2
Bipolaris oryzae	n/a	TG12bL2	GCA_001675385.1	NCBI	31.52	1,135	75	10,182	35	98.5	50.9	4.0
Bipolaris sorokiniana	n/a	BRIP10943a	GCA_008452735.1	NCBI	36.92	22	2,135	10,542	40	98.9	49.5	17.1
Bipolaris sorokiniana	n/a	BRIP27492a	GCA_008452725.1	NCBI	35.24	19	2,129	10,492	38	98.8	49.4	13.7
Bipolaris sorokiniana	n/a	BS112	GCA_004329375.1	NCBI	37.38	43	2,115	10,661	40	99.0	49.4	16.4
Bipolaris sorokiniana	n/a	ND90Pr	GCA_000338995.1	NCBI	34.41	154	1,789	10,251	41	98.9	49.8	8.8
Bipolaris sorokiniana	n/a	Shoemaker	GCA_013416765.1	NCBI	34.33	96	1,586	10,287	36	99.0	49.9	7.8
Bipolaris victoriae	n/a	FI3	GCA_000527765.2	NCBI	33.97	21	2,462	10,494	40	98.9	49.8	8.0
Bipolaris zeicola (Cochliobolus carbonum)	n/a	26-R-13	GCA_000523435.1	NCBI	31.27	844	110	10,245	50	98.8	50.8	2.5
Bipolaris zeicola (Cochliobolus carbonum)	n/a	GZL1	GCA_016906865.1	NCBI	36.14	23	2,045	10,528	47	98.8	50.6	15.4
Curvularia geniculata	n/a	P1	GCA_016162275.1	NCBI	32.92	574	248	10,371	33	98.3	50.7	7.2
Curvularia geniculata	n/a	W3	GCA_002982235.1	NCBI	33.54	737	227	10,475	36	99.0	50.6	7.8
Curvularia lunata	n/a	CX-3	GCA_000743335.1	NCBI	35.49	327	788	10,256	30	98.5	50.2	9.1
Curvularia lunata	n/a	W3	GCA_005212705.1	NCBI	33.54	737	227	10,468	36	99.0	50.6	7.8
Curvularia lunata (Cochliobolus lunatus)	n/a	m118	Coclu2	JGI	31.17	171	1,531	10,141	36	99.0	50.9	3.2
Curvularia papendorfii	n/a	UM226	GCA_000817285.1	NCBI	33.38	374	146	10,305	31	94.8	50.7	8.2
Curvularia sp	n/a	IFB-Z10	GCA_002161795.1	NCBI	33.01	136	1,950	10,483	33	98.6	50.5	7.4
Decorospora gaudefroyi	n/a	Decga1	Decga1	JGI	30.56	699	150	10,110	21	99.2	52.2	7.4
Decorospora gaudefroyi	n/a	P77	GCA_010015605.1	NCBI	30.56	699	150	10,083	21	98.8	52.2	7.4
Paradendryphiella salina	n/a	PS123737	GCA_900634815.1	NCBI	27.33	1,686	33	9,168	21	98.2	52.1	3.3
Pyrenophora graminea	n/a	CBS336.29	GCA_012365135.1	NCBI	32.55	2,965	28	10,542	53	97.5	50.7	5.2
Pyrenophora seminiperda	n/a	CCB06	GCA_000465215.2	NCBI	32.54	54	1,474	10,141	29	98.4	49.9	7.9
Pyrenophora teres	n/a	0-1	GCA_006112615.1	NCBI	46.51	55	4,380	10,680	70	98.2	46.6	33.6
Pyrenophora teres	n/a	15A	GCA_008086755.1	NCBI	45.32	119	2,996	11,054	79	98.2	47.8	31.4
Pyrenophora teres	n/a	6A	GCA_008086725.1	NCBI	48.62	48	4,076	10,659	79	97.6	45.7	36.9
Pyrenophora teres	n/a	BB25	GCA_008086785.1	NCBI	51.27	145	3,370	11,074	85	97.6	45.6	39.5
Pyrenophora teres	n/a	DEN2.6	GCA_014334755.1	NCBI	42.68	12	4,078	10,670	45	99.0	45.9	28.3
Pyrenophora teres	n/a	FGOB10Ptm-1	GCA_014334795.1	NCBI	38.17	12	3,415	10,527	45	99.0	45.7	19.8
Pyrenophora teres	n/a	FGOH04Ptt-21	GCA_008086845.1	NCBI	49.65	39	4,129	10,717	72	98.6	50.9	37.6
Pyrenophora teres	n/a	HRS9122	GCA_009728645.1	NCBI	47.98	44	3,214	10,632	60	98.5	45.8	35.7
Pyrenophora teres	n/a	HRS9139	GCA_009728635.1	NCBI	50.90	91	3,168	10,656	36	98.4	45.4	39.4

Pyrenophora teres	n/a	NB29	GCA_009728665.1	NCBI	50.12	55	3,271	10,891	37	98.9	45.6	38.6
Pyrenophora teres	n/a	NB73	GCA_009728655.1	NCBI	48.03	43	3,166	10,950	39	98.6	45.9	35.7
Pyrenophora teres	n/a	NB85	GCA_009728675.1	NCBI	49.03	47	3,319	10,758	58	98.8	45.7	37.5
Pyrenophora teres	n/a	NZKF2	GCA_014334775.1	NCBI	42.57	12	3,761	10,805	45	98.8	46.2	27.7
Pyrenophora teres	n/a	P-A14	GCA_014334815.1	NCBI	42.03	12	3,807	10,747	43	98.2	45.9	27.4
Pyrenophora teres	n/a	Pyrtt1	Pyrtt1	JGI	32.52	2,971	37	10,998	63	98.3	47.8	7.6
Pyrenophora teres	n/a	SG1	GCA_900231935.2	NCBI	41.28	33	3,641	10,442	47	98.8	46.9	26.1
Pyrenophora teres	n/a	W1-1	GCA_900232045.2	NCBI	53.08	57	4,721	10,577	82	98.7	45.2	39.6
Pyrenophora tritici-repentis	n/a	134	GCA_003231325.1	NCBI	33.57	2,007	66	10,867	36	99.0	50.9	7.2
Pyrenophora tritici-repentis	n/a	239	GCA_003231365.1	NCBI	34.06	1,957	67	10,859	36	98.9	50.9	7.3
Pyrenophora tritici-repentis	n/a	5213	GCA_003231345.1	NCBI	33.75	1,898	67	10,915	37	98.8	50.9	7.1
Pyrenophora tritici-repentis	n/a	11137	GCA_003231355.1	NCBI	33.63	1,720	75	10,869	36	98.8	50.9	5.9
Pyrenophora tritici-repentis	n/a	86-124-2	GCA_003231425.2	NCBI	34.15	2,486	61	11,203	35	99.0	51.1	6.8
Pyrenophora tritici-repentis	n/a	ARCrossB10	GCA_018492725.1	NCBI	40.11	142	687	11,221	38	98.0	50.7	22.3
Pyrenophora tritici-repentis	n/a	DW5	GCA_003231415.2	NCBI	40.87	60	3,134	11,172	36	99.0	50.7	23.9
Pyrenophora tritici-repentis	n/a	M4	GCA_003171515.2	NCBI	40.92	41	3,658	11,220	37	98.5	50.7	23.5
Pyrenophora tritici-repentis	n/a	Pt-1C-BFP	GCA_000149985.1	NCBI	38.00	48	1,986	11,036	40	98.2	51.0	17.0
Pyrenophora tritici-repentis	n/a	Pyrtr1	Pyrtr1	JGI	37.84	47	1,986	11,214	40	98.2	50.7	17.0
Pyrenophora tritici-repentis	n/a	V0001	GCA_008692205.1	NCBI	40.41	33	3,422	10,916	37	98.9	50.9	22.4
Setosphaeria rostrata (Exserohilum rostratum)	n/a	ER1	GCA_019453395.1	NCBI	35.19	29	1,787	10,677	50	97.8	50.6	5.6
Setosphaeria turcica	n/a	NY001	Settur3	JGI	38.42	489	235	10,320	50	98.8	51.9	13.4
Stemphylium lycopersici	n/a	CIDEFI212	GCA_003268315.1	NCBI	34.16	599	280	10,778	34	98.7	51.4	6.9
Stemphylium lycopersici	n/a	CIDEFI213	GCA_003268335.1	NCBI	35.00	787	185	10,617	36	98.8	51.2	7.8
Stemphylium lycopersici	n/a	CIDEFI-216	Stely1	JGI	35.17	414	498	10,687	31	98.7	50.8	9.8
Stemphylium vesicarium	n/a	173-1a-13FI1M3	GCA_004380135.1	NCBI	38.66	1,127	168	11,907	37	99.0	50.7	12.6
Stemphylium vesicarium	n/a	On16-391	GCA_008271585.1	NCBI	38.69	2,391	70	12,201	43	98.9	51.1	14.2
Stemphylium vesicarium	n/a	On16-63	GCA_008271615.1	NCBI	40.00	1,711	119	12,195	38	98.8	50.7	16.0

Species whose names have been updated are indicated with their previous species names in parentheses. Asterisks indicate Alternaria strains that were identified to section only, not to species. For Source: DET = Dettman laboratory; JGI = Joint Genome Institute; NCBI = National Centre for Biotechnology Information. Bolded rows indicate newly generated genomes being released here.

Table S2 List of programs and versions used in this study.

**Program**	**Version**
antiSMASH	6.0
AUGUSTUS	3.3.3
bamtools	2.5.1
bedtools	2.30.0
BiG-SCAPE	1.1.2
blat	36
clinker	0.0.30
CodingQuarry	2
diamond	2.0.8
emapper.py	2.1.3
ete3	3.1.2
EvidenceModeler	1.1.1
exonerate	2.4.0
Funannotate	1.8.7
GeneMark-ES/ET	4.65_lic
glimmerhmm	3.0.4
gmap	11/15/2017
hisat2	2.2.1
HMMER	3.3.2
kallisto	0.46.1
mafft	v7.475
minimap2	2.18-r1015
Orthofinder	2.5.4
PASA	2.4.1
proteinortho	6.0.30
Python	3.7.10
QUAST	0.38
salmon	0.14.1
samtools	1.1
signalp	5.0b
snap	7/28/2006
stringtie	2.1.5
tantan	26
trimAl	1.4.rev15
trimmomatic	0.39
Trinity	2.8.5
tRNAscan-SE	2.0.7

Table S3 Summary of gene prediction results from optimization trials for <funannotate predict>.

**Trial**	**Species**	**Reported # genes (NCBI)**	***Ab initio*, default weightings**	***Ab initio*, snap:0**	***Alternaria* trained, default weightings**	***Alternaria* trained, snap:0**	***Alternaria* trained, snap:0, transcript evidence**	***Alternaria* trained, Coding Quarry, PASA**	***Decorospora* trained, snap:0, transcript evidence**
1	*A. alternaria* SRC1lrK2f	13,466	10,941	-	11,089	12,093	12,234	13,372	-
2	*A. tenuissima* FERA1166	13,566	11,534	-	-	-	12,729	FAILED	-
3	*A. gaisen* FERA650	13,150	11,220	-	-	-	12,386	FAILED	-
4	*D. gaudefroyi* P77	11,835	-	10,294	-	-	10,246	-	10,631

Table S4 Numbers of genomes (in brackets) included in analyses, by taxonomic group.

**Family (*n*)**	**Genus (*n*)**	**Alternaria Section (*n*)**
Pleosporaceae (187)	*Alternaria* (123)	*Alternaria* (86)
		brassicae (1) monotypic lineage
		Brassicicola (3)
		Embellisia (1)
		Infectoriae (10)
		Porri (10)
		Pseudoalternaria (5)
		Pseudoulocladium (1)
		Ulocladioides (5)
		Ulocladium (1)
	Bipolaris (16)	
	Curvularia (7)	
	Decorospora (2)	
	Paradendryphiella (1)	
	Pyrenophora (30)	
	Setosphaeria (2)	
	Stemphylium (6)	

Table S5 Composition of higher-level BGC classes.

**Higher-Level BGC Class**	**Included antiSMASH Classes**
NRPS	NAPAA
	NRPS
	NRPS,indole
	NRPS,terpene
	NRPS,NRPS-like
	NRPS-like
	NRPS-like,indole
	NRPS-like,terpene
PKSI	T1PKS
	T1PKS,indole
	T1PKS,terpene
PKS-NRPS Hybrids	NRPS,T1PKS
	NRPS,NRPS-like,T1PKS
	NRPS-like,T1PKS
	NRPS-like,T1PKS,betalactone
	NRPS-like,T1PKS,indole
PKSIII	T3PKS
Terpene	terpene
Others	Betalactone
	Fungal-RiPP
	Indole

Table S6 Prevalence of all 548 GCFs across 187 taxa divided into four groups.

**GCF class**	**GCF Number**	**% *Alternaria* section *Alternaria* (n=86)**	**% *Alternaria* section *Infectoriae/ Pseudoalternaria* (n=15)**	**% "other" *Alternaria* (n=22)**	**% "other" *Pleosporaceae* (n=64)**	**Predicted compound, MiBIG accession**
NRPS	1	0.0	0.0	9.1	37.5	
NRPS	2	98.8	100.0	90.9	98.4	
NRPS	3	96.5	100.0	100.0	100.0	
NRPS	4	100.0	100.0	90.9	98.4	
NRPS	5	98.8	100.0	100.0	89.1	
NRPS	6	100.0	100.0	86.4	89.1	dimethylcoprogen, BGC0001249
NRPS	7	98.8	100.0	95.5	84.4	
NRPS	8	98.8	100.0	100.0	45.3	
NRPS	9	100.0	100.0	68.2	35.9	
NRPS	10	100.0	100.0	95.5	18.8	
NRPS	11	97.7	0.0	63.6	18.8	
NRPS	12	79.1	0.0	36.4	18.8	
NRPS	13	0.0	0.0	0.0	46.9	apicidin, BGC0000304
**NRPS**	**14**	**20.9**	**0.0**	**9.1**	**0.0**	
NRPS	15	0.0	0.0	0.0	39.1	
NRPS	16	0.0	0.0	0.0	26.6	
NRPS	17	0.0	0.0	0.0	21.9	
NRPS	18	18.6	0.0	0.0	1.6	
NRPS	19	0.0	0.0	0.0	17.2	
NRPS	20	0.0	0.0	0.0	23.4	
NRPS	21	0.0	0.0	0.0	21.9	
NRPS	22	0.0	0.0	0.0	21.9	
**NRPS**	**23**	**16.3**	**0.0**	**0.0**	**0.0**	
NRPS	24	14.0	0.0	0.0	1.6	
NRPS	25	0.0	0.0	0.0	18.8	
NRPS	26	0.0	46.7	0.0	7.8	

NRPS	27	0.0	0.0	0.0	18.8	
NRPS	28	0.0	0.0	0.0	15.6	
NRPS	29	0.0	0.0	0.0	14.1	
NRPS	30	0.0	0.0	0.0	14.1	
NRPS	31	0.0	0.0	0.0	12.5	
NRPS	32	0.0	0.0	0.0	12.5	
NRPS	33	0.0	0.0	0.0	10.9	
NRPS	34	0.0	0.0	0.0	10.9	
NRPS	35	0.0	0.0	0.0	9.4	
**NRPS**	**36**	**0.0**	**0.0**	**27.3**	**0.0**	
**NRPS**	**37**	**7.0**	**0.0**	**0.0**	**0.0**	**AM-toxin, BGC0001261**
NRPS	38	0.0	0.0	0.0	9.4	
**NRPS**	**39**	**0.0**	**0.0**	**27.3**	**0.0**	
NRPS	40	0.0	0.0	0.0	7.8	
NRPS	41	0.0	0.0	0.0	7.8	
NRPS	42	0.0	0.0	0.0	4.7	
NRPS	43	0.0	0.0	0.0	7.8	
NRPS	44	0.0	0.0	0.0	7.8	
NRPS	45	0.0	0.0	0.0	7.8	
NRPS	46	0.0	0.0	0.0	6.3	
**NRPS**	**47**	**0.0**	**0.0**	**18.2**	**0.0**	
**NRPS**	**48**	**4.7**	**0.0**	**0.0**	**0.0**	
NRPS	49	0.0	0.0	0.0	6.3	
NRPS	50	0.0	0.0	0.0	6.3	
NRPS	51	0.0	0.0	0.0	6.3	
NRPS	52	0.0	0.0	0.0	6.3	
NRPS	53	0.0	0.0	0.0	6.3	
**NRPS**	**54**	**1.2**	**0.0**	**13.6**	**0.0**	
NRPS	55	0.0	0.0	0.0	6.3	
NRPS	56	0.0	0.0	0.0	6.3	

NRPS	57	1.2	0.0	0.0	4.7	
NRPS	58	0.0	0.0	0.0	6.3	
**NRPS**	**59**	**0.0**	**0.0**	**13.6**	**0.0**	
NRPS	60	0.0	0.0	0.0	4.7	
NRPS	61	0.0	0.0	0.0	1.6	
NRPS	62	0.0	0.0	0.0	4.7	
NRPS	63	0.0	0.0	4.5	1.6	HC-toxin, BGC0001166
NRPS	64	0.0	0.0	0.0	3.1	
NRPS	65	0.0	0.0	0.0	4.7	
NRPS	66	0.0	0.0	0.0	4.7	
NRPS	67	0.0	0.0	0.0	4.7	
NRPS	68	0.0	0.0	0.0	4.7	
**NRPS**	**69**	**3.5**	**0.0**	**0.0**	**0.0**	
NRPS	70	0.0	0.0	0.0	4.7	
NRPS	71	0.0	0.0	0.0	4.7	
NRPS	72	0.0	0.0	0.0	4.7	
NRPS	73	0.0	0.0	0.0	3.1	
**NRPS**	**74**	**0.0**	**0.0**	**9.1**	**0.0**	
NRPS	75	0.0	0.0	0.0	3.1	
**NRPS**	**76**	**2.3**	**0.0**	**0.0**	**0.0**	**altersetin/equisetin, BGC0001255**
NRPS	77	0.0	0.0	0.0	3.1	
**NRPS**	**78**	**0.0**	**0.0**	**9.1**	**0.0**	
NRPS	79	0.0	0.0	0.0	3.1	
**NRPS**	**80**	**2.3**	**0.0**	**0.0**	**0.0**	
NRPS	81	0.0	0.0	0.0	1.6	
NRPS	82	0.0	0.0	0.0	3.1	
**NRPS**	**83**	**0.0**	**0.0**	**9.1**	**0.0**	
NRPS	84	0.0	0.0	0.0	1.6	
**NRPS**	**85**	**0.0**	**0.0**	**9.1**	**0.0**	

**NRPS**	**86**	**0.0**	**0.0**	**9.1**	**0.0**	**KK-1, BGC0001636**
NRPS	87	0.0	0.0	0.0	3.1	
NRPS	88	0.0	0.0	0.0	1.6	
NRPS	89	0.0	0.0	0.0	3.1	
NRPS	90	0.0	0.0	0.0	1.6	
NRPS	91	0.0	0.0	0.0	3.1	
NRPS	92	0.0	0.0	0.0	3.1	
**NRPS**	**93**	**2.3**	**0.0**	**0.0**	**0.0**	
NRPS	94	0.0	0.0	0.0	3.1	
NRPS	95	0.0	0.0	0.0	3.1	
NRPS	96	0.0	0.0	0.0	3.1	
NRPS	97	0.0	0.0	0.0	3.1	
**NRPS**	**98**	**1.2**	**0.0**	**0.0**	**0.0**	
**NRPS**	**99**	**1.2**	**0.0**	**0.0**	**0.0**	**altersetin/equisetin, BGC0001255**
**NRPS**	**100**	**1.2**	**0.0**	**0.0**	**0.0**	
**NRPS**	**101**	**1.2**	**0.0**	**0.0**	**0.0**	
**NRPS**	**102**	**1.2**	**0.0**	**0.0**	**0.0**	
**NRPS**	**103**	**1.2**	**0.0**	**0.0**	**0.0**	
**NRPS**	**104**	**1.2**	**0.0**	**0.0**	**0.0**	
**NRPS**	**105**	**1.2**	**0.0**	**0.0**	**0.0**	
**NRPS**	**106**	**1.2**	**0.0**	**0.0**	**0.0**	
**NRPS**	**107**	**1.2**	**0.0**	**0.0**	**0.0**	
**NRPS**	**108**	**1.2**	**0.0**	**0.0**	**0.0**	
**NRPS**	**109**	**1.2**	**0.0**	**0.0**	**0.0**	
**NRPS**	**110**	**0.0**	**0.0**	**4.5**	**0.0**	
**NRPS**	**111**	**0.0**	**0.0**	**4.5**	**0.0**	
**NRPS**	**112**	**0.0**	**0.0**	**4.5**	**0.0**	
**NRPS**	**113**	**1.2**	**0.0**	**0.0**	**0.0**	
**NRPS**	**114**	**0.0**	**0.0**	**4.5**	**0.0**	

**NRPS**	**115**	**0.0**	**0.0**	**4.5**	**0.0**	
**NRPS**	**116**	**0.0**	**0.0**	**4.5**	**0.0**	
**NRPS**	**117**	**0.0**	**0.0**	**4.5**	**0.0**	
**NRPS**	**118**	**0.0**	**0.0**	**4.5**	**0.0**	
**NRPS**	**119**	**0.0**	**0.0**	**4.5**	**0.0**	
**NRPS**	**120**	**0.0**	**0.0**	**4.5**	**0.0**	
**NRPS**	**121**	**0.0**	**0.0**	**4.5**	**0.0**	
**NRPS**	**122**	**0.0**	**0.0**	**4.5**	**0.0**	
**NRPS**	**123**	**1.2**	**0.0**	**0.0**	**0.0**	
**NRPS**	**124**	**1.2**	**0.0**	**0.0**	**0.0**	
**NRPS**	**125**	**1.2**	**0.0**	**0.0**	**0.0**	
**NRPS**	**126**	**1.2**	**0.0**	**0.0**	**0.0**	
**NRPS**	**127**	**0.0**	**0.0**	**4.5**	**0.0**	
**NRPS**	**128**	**0.0**	**0.0**	**4.5**	**0.0**	
**NRPS**	**129**	**0.0**	**0.0**	**4.5**	**0.0**	
**NRPS**	**130**	**0.0**	**0.0**	**4.5**	**0.0**	
**NRPS**	**131**	**0.0**	**0.0**	**4.5**	**0.0**	
**NRPS**	**132**	**0.0**	**0.0**	**4.5**	**0.0**	
**NRPS**	**133**	**0.0**	**6.7**	**0.0**	**0.0**	
**NRPS**	**134**	**0.0**	**0.0**	**4.5**	**0.0**	
**NRPS**	**135**	**0.0**	**0.0**	**4.5**	**0.0**	
**NRPS**	**136**	**0.0**	**0.0**	**4.5**	**0.0**	
**NRPS**	**137**	**0.0**	**0.0**	**4.5**	**0.0**	
**NRPS**	**138**	**0.0**	**0.0**	**4.5**	**0.0**	
**NRPS**	**139**	**0.0**	**0.0**	**4.5**	**0.0**	
NRPS	140	0.0	0.0	0.0	1.6	
NRPS	141	0.0	0.0	0.0	1.6	
NRPS	142	0.0	0.0	0.0	1.6	
NRPS	143	0.0	0.0	0.0	1.6	
NRPS	144	0.0	0.0	0.0	1.6	

NRPS	145	0.0	0.0	0.0	1.6	
NRPS	146	0.0	0.0	0.0	1.6	
NRPS	147	0.0	0.0	0.0	1.6	
NRPS	148	0.0	0.0	0.0	1.6	
NRPS	149	0.0	0.0	0.0	1.6	
NRPS	150	0.0	0.0	0.0	1.6	
NRPS	151	0.0	0.0	0.0	1.6	
NRPS	152	0.0	0.0	0.0	1.6	
NRPS	153	0.0	0.0	0.0	1.6	
NRPS	154	0.0	0.0	0.0	1.6	
NRPS	155	0.0	0.0	0.0	1.6	
NRPS	156	0.0	0.0	0.0	1.6	
NRPS	157	0.0	0.0	0.0	1.6	
NRPS	158	0.0	0.0	0.0	1.6	
NRPS	159	0.0	0.0	0.0	1.6	
NRPS	160	0.0	0.0	0.0	1.6	
NRPS	161	0.0	0.0	0.0	1.6	
NRPS	162	0.0	0.0	0.0	1.6	
NRPS	163	0.0	0.0	0.0	1.6	
NRPS	164	0.0	0.0	0.0	1.6	
NRPS	165	0.0	0.0	0.0	1.6	
NRPS	166	0.0	0.0	0.0	1.6	
NRPS	167	0.0	0.0	0.0	1.6	
NRPS	168	0.0	0.0	0.0	1.6	
NRPS	169	0.0	0.0	0.0	1.6	phomasetin, BGC0001738
NRPS	170	0.0	0.0	0.0	1.6	
NRPS	171	0.0	0.0	0.0	1.6	
NRPS	172	0.0	0.0	0.0	1.6	
NRPS	173	0.0	0.0	0.0	1.6	
NRPS	174	0.0	0.0	0.0	1.6	

NRPS	175	0.0	0.0	0.0	1.6	
NRPS	176	0.0	0.0	0.0	1.6	
NRPS	177	0.0	0.0	0.0	1.6	
NRPS	178	0.0	0.0	0.0	1.6	
NRPS	179	0.0	0.0	0.0	1.6	
NRPS	180	0.0	0.0	0.0	1.6	
NRPS	181	0.0	0.0	0.0	1.6	
NRPS	182	0.0	0.0	0.0	1.6	
NRPS	183	0.0	0.0	0.0	1.6	
NRPS	184	0.0	0.0	0.0	1.6	
NRPS	185	0.0	0.0	0.0	1.6	phomasetin, BGC0001738
NRPS	186	0.0	0.0	0.0	1.6	
NRPS	187	0.0	0.0	0.0	1.6	
NRPS	188	0.0	0.0	0.0	1.6	
NRPS	189	0.0	0.0	0.0	1.6	
NRPS	190	0.0	0.0	0.0	1.6	
NRPS	191	0.0	0.0	0.0	1.6	
NRPS	192	0.0	0.0	0.0	1.6	
NRPS	193	0.0	0.0	0.0	1.6	
NRPS	194	0.0	0.0	0.0	1.6	
NRPS	195	0.0	0.0	0.0	1.6	
NRPS	196	0.0	0.0	0.0	1.6	
NRPS	197	0.0	0.0	0.0	1.6	
NRPS	198	0.0	0.0	0.0	1.6	
NRPS	199	0.0	0.0	0.0	1.6	
NRPS	200	0.0	0.0	0.0	1.6	
NRPS	201	0.0	0.0	0.0	1.6	
NRPS	202	0.0	0.0	0.0	1.6	
NRPS	203	0.0	0.0	0.0	1.6	
NRPS	204	0.0	0.0	0.0	1.6	

NRPS	205	0.0	0.0	0.0	1.6	
NRPS	206	0.0	0.0	0.0	1.6	
NRPS	207	0.0	0.0	0.0	1.6	
NRPS	208	0.0	0.0	0.0	1.6	
NRPS	209	0.0	0.0	0.0	1.6	
NRPS	210	0.0	0.0	0.0	1.6	
NRPS	211	0.0	0.0	0.0	1.6	
NRPS	212	0.0	0.0	0.0	1.6	
NRPS	213	0.0	0.0	0.0	1.6	
NRPS	214	0.0	0.0	0.0	1.6	
NRPS	215	0.0	0.0	0.0	1.6	
NRPS	216	0.0	0.0	0.0	1.6	
NRPS	217	0.0	0.0	0.0	1.6	
NRPS	218	0.0	0.0	0.0	1.6	
NRPS	219	0.0	0.0	0.0	1.6	
NRPS	220	0.0	0.0	0.0	1.6	
NRPS	221	0.0	0.0	0.0	1.6	
NRPS	222	0.0	0.0	0.0	1.6	
NRPS	223	0.0	0.0	0.0	1.6	
NRPS	224	0.0	0.0	0.0	1.6	
NRPS	225	0.0	0.0	0.0	1.6	
NRPS	226	0.0	0.0	0.0	1.6	
NRPS	227	0.0	0.0	0.0	1.6	
NRPS	228	0.0	0.0	0.0	1.6	
NRPS	229	0.0	0.0	0.0	1.6	
NRPS	230	0.0	0.0	0.0	1.6	
NRPS	231	0.0	0.0	0.0	1.6	
NRPS	232	0.0	0.0	0.0	1.6	
NRPS	233	0.0	0.0	0.0	1.6	
NRPS	234	0.0	0.0	0.0	1.6	

NRPS	235	0.0	0.0	0.0	1.6	
NRPS	236	0.0	0.0	0.0	1.6	
NRPS	237	0.0	0.0	0.0	1.6	
NRPS	238	0.0	0.0	0.0	1.6	
NRPS	239	0.0	0.0	0.0	1.6	
NRPS	240	0.0	0.0	0.0	1.6	
NRPS	241	0.0	0.0	0.0	1.6	
NRPS	242	0.0	0.0	0.0	1.6	
NRPS	243	0.0	0.0	0.0	1.6	
NRPS	244	0.0	0.0	0.0	1.6	
NRPS	245	0.0	0.0	0.0	1.6	
NRPS	246	0.0	0.0	0.0	1.6	
NRPS	247	0.0	0.0	0.0	1.6	
NRPS	248	0.0	0.0	0.0	1.6	
NRPS	249	0.0	0.0	0.0	1.6	
NRPS	250	0.0	0.0	0.0	1.6	
NRPS	251	0.0	0.0	0.0	1.6	
NRPS	252	0.0	0.0	0.0	1.6	
NRPS	253	0.0	0.0	0.0	1.6	
NRPS	254	0.0	0.0	0.0	1.6	
NRPS	255	0.0	0.0	0.0	1.6	
NRPS	256	0.0	0.0	0.0	1.6	
NRPS	257	0.0	0.0	0.0	1.6	
NRPS	258	0.0	0.0	0.0	1.6	
NRPS	259	0.0	0.0	0.0	1.6	
PKS-NRPS Hybrids	1	86.0	100.0	40.9	32.8	
**PKS-NRPS Hybrids**	**2**	**84.9**	**0.0**	**0.0**	**0.0**	**altersetin/equisetin, BGC0001255**
**PKS-NRPS Hybrids**	**3**	**75.6**	**0.0**	**0.0**	**0.0**	

PKS-NRPS Hybrids	4	0.0	0.0	13.6	28.1	phomasetin, BGC0001738
**PKS-NRPS Hybrids**	**5**	**19.8**	**0.0**	**4.5**	**0.0**	
PKS-NRPS Hybrids	6	0.0	0.0	0.0	17.2	
PKS-NRPS Hybrids	7	0.0	0.0	0.0	26.6	
PKS-NRPS Hybrids	8	0.0	0.0	0.0	25.0	
PKS-NRPS Hybrids	9	0.0	0.0	0.0	25.0	curvupallide-B, BGC0001563
**PKS-NRPS Hybrids**	**10**	**0.0**	**100.0**	**0.0**	**0.0**	
PKS-NRPS Hybrids	11	0.0	0.0	0.0	21.9	
PKS-NRPS Hybrids	12	0.0	0.0	0.0	21.9	
PKS-NRPS Hybrids	13	0.0	0.0	0.0	20.3	
PKS-NRPS Hybrids	14	0.0	0.0	0.0	18.8	
PKS-NRPS Hybrids	15	0.0	0.0	45.5	1.6	
PKS-NRPS Hybrids	16	0.0	0.0	0.0	10.9	
PKS-NRPS Hybrids	17	0.0	0.0	22.7	1.6	BII-rafflesfungin, BGC0001966
PKS-NRPS Hybrids	18	0.0	0.0	0.0	9.4	
PKS-NRPS Hybrids	19	0.0	0.0	0.0	7.8	
PKS-NRPS Hybrids	20	0.0	0.0	0.0	7.8	
PKS-NRPS Hybrids	21	0.0	0.0	0.0	7.8	dimethylcoprogen, BGC0001249
PKS-NRPS Hybrids	22	0.0	0.0	0.0	6.3	

**PKS-NRPS Hybrids**	**23**	**3.5**	**0.0**	**0.0**	**0.0**	**ACT-Toxin II, BGC0001254**
PKS-NRPS Hybrids	24	0.0	0.0	0.0	4.7	
**PKS-NRPS Hybrids**	**25**	**0.0**	**0.0**	**13.6**	**0.0**	**dimethylcoprogen, BGC0001249**
**PKS-NRPS Hybrids**	**26**	**2.3**	**0.0**	**0.0**	**0.0**	
PKS-NRPS Hybrids	27	0.0	0.0	0.0	3.1	
PKS-NRPS Hybrids	28	0.0	0.0	0.0	3.1	
PKS-NRPS Hybrids	29	0.0	0.0	0.0	3.1	
**PKS-NRPS Hybrids**	**30**	**2.3**	**0.0**	**0.0**	**0.0**	
PKS-NRPS Hybrids	31	0.0	0.0	0.0	3.1	dimethylcoprogen, BGC0001249
**PKS-NRPS Hybrids**	**32**	**2.3**	**0.0**	**0.0**	**0.0**	
PKS-NRPS Hybrids	33	0.0	0.0	0.0	3.1	
**PKS-NRPS Hybrids**	**34**	**1.2**	**0.0**	**0.0**	**0.0**	
**PKS-NRPS Hybrids**	**35**	**0.0**	**0.0**	**4.5**	**0.0**	
**PKS-NRPS Hybrids**	**36**	**0.0**	**0.0**	**4.5**	**0.0**	
**PKS-NRPS Hybrids**	**37**	**0.0**	**0.0**	**4.5**	**0.0**	
**PKS-NRPS Hybrids**	**38**	**0.0**	**0.0**	**4.5**	**0.0**	
**PKS-NRPS Hybrids**	**39**	**0.0**	**0.0**	**4.5**	**0.0**	
**PKS-NRPS Hybrids**	**40**	**0.0**	**6.7**	**0.0**	**0.0**	
PKS-NRPS Hybrids	41	0.0	0.0	0.0	1.6	

PKS-NRPS Hybrids	42	0.0	0.0	0.0	1.6	
PKS-NRPS Hybrids	43	0.0	0.0	0.0	1.6	
PKS-NRPS Hybrids	44	0.0	0.0	0.0	1.6	
PKS-NRPS Hybrids	45	0.0	0.0	0.0	1.6	
PKS-NRPS Hybrids	46	0.0	0.0	0.0	1.6	
PKS-NRPS Hybrids	47	0.0	0.0	0.0	1.6	
PKS-NRPS Hybrids	48	0.0	0.0	0.0	1.6	
PKS-NRPS Hybrids	49	0.0	0.0	0.0	1.6	
PKS-NRPS Hybrids	50	0.0	0.0	0.0	1.6	
PKS-NRPS Hybrids	51	0.0	0.0	0.0	1.6	
PKS-NRPS Hybrids	52	0.0	0.0	0.0	1.6	
PKS-NRPS Hybrids	53	0.0	0.0	0.0	1.6	
PKS-NRPS Hybrids	54	0.0	0.0	0.0	1.6	
PKS-NRPS Hybrids	55	0.0	0.0	0.0	1.6	
PKS-NRPS Hybrids	56	0.0	0.0	0.0	1.6	
PKS-NRPS Hybrids	57	0.0	0.0	0.0	1.6	
PKS-NRPS Hybrids	58	0.0	0.0	0.0	1.6	
PKS-NRPS Hybrids	59	0.0	0.0	0.0	1.6	
PKS-NRPS Hybrids	60	0.0	0.0	0.0	1.6	curvupallide-B, BGC0001563

PKS-NRPS Hybrids	61	0.0	0.0	0.0	1.6	
PKS-NRPS Hybrids	62	0.0	0.0	0.0	1.6	
PKS-NRPS Hybrids	63	0.0	0.0	0.0	1.6	
PKS-NRPS Hybrids	64	0.0	0.0	0.0	1.6	
PKS-NRPS Hybrids	65	0.0	0.0	0.0	1.6	
PKS-NRPS Hybrids	66	0.0	0.0	0.0	1.6	
PKS-NRPS Hybrids	67	0.0	0.0	0.0	1.6	
PKS-NRPS Hybrids	68	0.0	0.0	0.0	1.6	
PKS-NRPS Hybrids	69	0.0	0.0	0.0	1.6	
PKS-NRPS Hybrids	70	0.0	0.0	0.0	1.6	
PKS-NRPS Hybrids	71	0.0	0.0	0.0	1.6	
PKS-NRPS Hybrids	72	0.0	0.0	0.0	1.6	
PKSI	1	98.8	100.0	90.9	100.0	melanin, BGC0001265 (has been retired)
PKSI	2	98.8	100.0	81.8	95.3	
PKSI	3	100.0	100.0	50.0	73.4	alternapyrone, BGC0000012
PKSI	4	94.2	100.0	31.8	23.4	
**PKSI**	**5**	**97.7**	**0.0**	**45.5**	**0.0**	**alternariol, BGC0001284**
PKSI	6	47.7	0.0	13.6	64.1	betaenone A, BGC0001264 (renamed BGC0001280)
**PKSI**	**7**	**90.7**	**0.0**	**0.0**	**0.0**	
PKSI	8	0.0	100.0	54.5	60.9	
PKSI	9	23.3	66.7	54.5	29.7	
PKSI	10	0.0	0.0	0.0	71.9	

PKSI	11	20.9	0.0	22.7	4.7	
**PKSI**	**12**	**32.6**	**0.0**	**13.6**	**0.0**	
PKSI	13	0.0	0.0	0.0	45.3	
PKSI	14	0.0	0.0	0.0	43.8	
PKSI	15	0.0	0.0	36.4	29.7	
PKSI	16	3.5	0.0	45.5	18.8	dehydrocurvularin, BGC0000045
PKSI	17	0.0	0.0	0.0	39.1	
PKSI	18	0.0	0.0	0.0	37.5	
PKSI	19	0.0	0.0	4.5	28.1	
PKSI	20	0.0	0.0	0.0	29.7	
PKSI	21	0.0	0.0	0.0	28.1	
PKSI	22	0.0	0.0	0.0	28.1	
PKSI	23	0.0	0.0	18.2	20.3	
**PKSI**	**24**	**19.8**	**0.0**	**0.0**	**0.0**	
PKSI	25	3.5	0.0	0.0	18.8	
PKSI	26	0.0	0.0	0.0	21.9	
**PKSI**	**27**	**0.0**	**100.0**	**0.0**	**0.0**	
**PKSI**	**28**	**0.0**	**100.0**	**0.0**	**0.0**	
**PKSI**	**29**	**0.0**	**93.3**	**0.0**	**0.0**	
PKSI	30	0.0	0.0	22.7	12.5	
PKSI	31	0.0	0.0	0.0	18.8	
PKSI	32	0.0	0.0	0.0	15.6	
PKSI	33	0.0	0.0	0.0	17.2	
PKSI	34	0.0	0.0	40.9	1.6	
PKSI	35	7.0	0.0	0.0	6.3	AK-toxin, BGC0001262<br>AF-toxin, BGC0000003
PKSI	36	0.0	0.0	0.0	15.6	
PKSI	37	0.0	0.0	0.0	14.1	
**PKSI**	**38**	**0.0**	**60.0**	**0.0**	**0.0**	
PKSI	39	0.0	0.0	0.0	12.5	

PKSI	40	0.0	0.0	0.0	12.5	
PKSI	41	0.0	0.0	0.0	12.5	
PKSI	42	0.0	0.0	13.6	6.3	
PKSI	43	0.0	0.0	0.0	10.9	
PKSI	44	0.0	0.0	0.0	10.9	
PKSI	45	0.0	0.0	0.0	9.4	
PKSI	46	0.0	0.0	0.0	9.4	
PKSI	47	0.0	0.0	0.0	9.4	
**PKSI**	**48**	**0.0**	**33.3**	**0.0**	**0.0**	
PKSI	49	0.0	0.0	0.0	7.8	
PKSI	50	0.0	0.0	0.0	7.8	
PKSI	51	0.0	0.0	0.0	7.8	
PKSI	52	0.0	0.0	0.0	7.8	
PKSI	53	0.0	0.0	0.0	7.8	
**PKSI**	**54**	**0.0**	**0.0**	**22.7**	**0.0**	
**PKSI**	**55**	**0.0**	**0.0**	**22.7**	**0.0**	
**PKSI**	**56**	**4.7**	**0.0**	**0.0**	**0.0**	
PKSI	57	0.0	0.0	0.0	4.7	
**PKSI**	**58**	**3.5**	**0.0**	**4.5**	**0.0**	**solanapyrone D, BGC0000146**
PKSI	59	0.0	0.0	0.0	6.3	
PKSI	60	0.0	0.0	0.0	6.3	
PKSI	61	0.0	0.0	0.0	6.3	
**PKSI**	**62**	**0.0**	**0.0**	**18.2**	**0.0**	
**PKSI**	**63**	**0.0**	**0.0**	**13.6**	**0.0**	
PKSI	64	0.0	0.0	0.0	4.7	
PKSI	65	0.0	0.0	0.0	4.7	
**PKSI**	**66**	**0.0**	**0.0**	**13.6**	**0.0**	
**PKSI**	**67**	**0.0**	**0.0**	**13.6**	**0.0**	**depudecin, BGC0000046**
PKSI	68	0.0	0.0	0.0	4.7	
**PKSI**	**69**	**3.5**	**0.0**	**0.0**	**0.0**	

PKSI	70	0.0	0.0	0.0	4.7	
**PKSI**	**71**	**3.5**	**0.0**	**0.0**	**0.0**	
**PKSI**	**72**	**1.2**	**0.0**	**9.1**	**0.0**	
PKSI	73	0.0	0.0	0.0	4.7	
**PKSI**	**74**	**0.0**	**0.0**	**9.1**	**0.0**	
PKSI	75	0.0	0.0	0.0	3.1	
**PKSI**	**76**	**0.0**	**0.0**	**9.1**	**0.0**	
PKSI	77	0.0	0.0	0.0	3.1	
PKSI	78	0.0	0.0	0.0	3.1	
PKSI	79	0.0	0.0	0.0	3.1	
PKSI	80	0.0	0.0	0.0	3.1	
PKSI	81	0.0	0.0	0.0	3.1	
PKSI	82	0.0	0.0	0.0	3.1	
PKSI	83	0.0	0.0	0.0	3.1	
PKSI	84	0.0	0.0	0.0	3.1	
PKSI	85	0.0	0.0	0.0	3.1	
PKSI	86	0.0	0.0	0.0	3.1	
PKSI	87	0.0	0.0	0.0	3.1	
PKSI	88	0.0	0.0	0.0	3.1	
PKSI	89	0.0	0.0	0.0	3.1	
**PKSI**	**90**	**0.0**	**0.0**	**9.1**	**0.0**	**solanapyrone D, BGC0000146**
PKSI	91	0.0	0.0	0.0	3.1	
PKSI	92	0.0	0.0	0.0	3.1	
PKSI	93	0.0	0.0	0.0	3.1	
PKSI	94	0.0	0.0	0.0	3.1	
**PKSI**	**95**	**1.2**	**0.0**	**0.0**	**0.0**	
**PKSI**	**96**	**1.2**	**0.0**	**0.0**	**0.0**	
**PKSI**	**97**	**1.2**	**0.0**	**0.0**	**0.0**	
**PKSI**	**98**	**0.0**	**0.0**	**4.5**	**0.0**	
**PKSI**	**99**	**0.0**	**0.0**	**4.5**	**0.0**	

**PKSI**	**100**	**0.0**	**0.0**	**4.5**	**0.0**	
**PKSI**	**101**	**0.0**	**0.0**	**4.5**	**0.0**	
**PKSI**	**102**	**0.0**	**0.0**	**4.5**	**0.0**	
**PKSI**	**103**	**0.0**	**0.0**	**4.5**	**0.0**	
**PKSI**	**104**	**0.0**	**0.0**	**4.5**	**0.0**	
**PKSI**	**105**	**1.2**	**0.0**	**0.0**	**0.0**	
**PKSI**	**106**	**0.0**	**0.0**	**4.5**	**0.0**	
**PKSI**	**107**	**0.0**	**0.0**	**4.5**	**0.0**	
**PKSI**	**108**	**0.0**	**0.0**	**4.5**	**0.0**	
**PKSI**	**109**	**0.0**	**0.0**	**4.5**	**0.0**	
**PKSI**	**110**	**0.0**	**0.0**	**4.5**	**0.0**	
**PKSI**	**111**	**1.2**	**0.0**	**0.0**	**0.0**	
**PKSI**	**112**	**1.2**	**0.0**	**0.0**	**0.0**	
**PKSI**	**113**	**1.2**	**0.0**	**0.0**	**0.0**	
**PKSI**	**114**	**0.0**	**0.0**	**4.5**	**0.0**	
**PKSI**	**115**	**0.0**	**0.0**	**4.5**	**0.0**	
**PKSI**	**116**	**0.0**	**0.0**	**4.5**	**0.0**	
**PKSI**	**117**	**0.0**	**0.0**	**4.5**	**0.0**	
PKSI	118	0.0	0.0	0.0	1.6	
PKSI	119	0.0	0.0	0.0	1.6	
PKSI	120	0.0	0.0	0.0	1.6	
PKSI	121	0.0	0.0	0.0	1.6	
PKSI	122	0.0	0.0	0.0	1.6	
PKSI	123	0.0	0.0	0.0	1.6	
PKSI	124	0.0	0.0	0.0	1.6	
PKSI	125	0.0	0.0	0.0	1.6	
PKSI	126	0.0	0.0	0.0	1.6	
PKSI	127	0.0	0.0	0.0	1.6	
PKSI	128	0.0	0.0	0.0	1.6	
PKSI	129	0.0	0.0	0.0	1.6	

PKSI	130	0.0	0.0	0.0	1.6	
PKSI	131	0.0	0.0	0.0	1.6	
PKSI	132	0.0	0.0	0.0	1.6	
PKSI	133	0.0	0.0	0.0	1.6	
PKSI	134	0.0	0.0	0.0	1.6	
PKSI	135	0.0	0.0	0.0	1.6	
PKSI	136	0.0	0.0	0.0	1.6	
PKSI	137	0.0	0.0	0.0	1.6	
PKSI	138	0.0	0.0	0.0	1.6	
PKSI	139	0.0	0.0	0.0	1.6	
PKSI	140	0.0	0.0	0.0	1.6	
PKSI	141	0.0	0.0	0.0	1.6	
PKSI	142	0.0	0.0	0.0	1.6	
PKSI	143	0.0	0.0	0.0	1.6	
PKSI	144	0.0	0.0	0.0	1.6	
PKSI	145	0.0	0.0	0.0	1.6	
PKSI	146	0.0	0.0	0.0	1.6	
PKSI	147	0.0	0.0	0.0	1.6	
PKSI	148	0.0	0.0	0.0	1.6	
PKSI	149	0.0	0.0	0.0	1.6	
PKSI	150	0.0	0.0	0.0	1.6	
PKSI	151	0.0	0.0	0.0	1.6	
PKSI	152	0.0	0.0	0.0	1.6	
PKSI	153	0.0	0.0	0.0	1.6	
PKSother	1	69.8	100.0	86.4	46.9	
PKSother	2	0.0	0.0	0.0	21.9	
PKSother	3	0.0	0.0	0.0	7.8	
PKSother	4	0.0	0.0	0.0	3.1	
**PKSother**	**5**	**1.2**	**0.0**	**0.0**	**0.0**	
PKSother	6	0.0	0.0	0.0	1.6	

PKSother	7	0.0	0.0	0.0	1.6	
PKSother	8	0.0	0.0	0.0	1.6	
**RiPPs**	**1**	**97.7**	**0.0**	**40.9**	**0.0**	
**RiPPs**	**2**	**0.0**	**60.0**	**0.0**	**0.0**	
RiPPs	3	0.0	0.0	0.0	12.5	
Terpene	1	100.0	100.0	100.0	98.4	
Terpene	2	100.0	100.0	100.0	98.4	
Terpene	3	98.8	100.0	81.8	95.3	
Terpene	4	73.3	100.0	72.7	85.9	
Terpene	5	33.7	13.3	45.5	31.3	
Terpene	6	34.9	0.0	0.0	21.9	
**Terpene**	**7**	**26.7**	**0.0**	**0.0**	**0.0**	
Terpene	8	0.0	0.0	0.0	28.1	
Terpene	9	0.0	0.0	0.0	23.4	
Terpene	10	0.0	0.0	0.0	15.6	
Terpene	11	0.0	0.0	0.0	10.9	
**Terpene**	**12**	**5.8**	**0.0**	**4.5**	**0.0**	
**Terpene**	**13**	**0.0**	**0.0**	**22.7**	**0.0**	
Terpene	14	0.0	0.0	0.0	4.7	
**Terpene**	**15**	**0.0**	**0.0**	**13.6**	**0.0**	
**Terpene**	**16**	**0.0**	**0.0**	**13.6**	**0.0**	**brassicicene C, BGC0000685**
Terpene	17	0.0	0.0	0.0	3.1	
Terpene	18	0.0	0.0	0.0	3.1	
**Terpene**	**19**	**2.3**	**0.0**	**0.0**	**0.0**	
**Terpene**	**20**	**2.3**	**0.0**	**0.0**	**0.0**	
Terpene	21	0.0	0.0	0.0	3.1	
Terpene	22	0.0	0.0	0.0	3.1	
**Terpene**	**23**	**0.0**	**0.0**	**9.1**	**0.0**	
Terpene	24	0.0	0.0	0.0	3.1	
Terpene	25	0.0	0.0	0.0	3.1	

Terpene	26	0.0	0.0	0.0	3.1	
**Terpene**	**27**	**1.2**	**0.0**	**0.0**	**0.0**	
**Terpene**	**28**	**1.2**	**0.0**	**0.0**	**0.0**	
**Terpene**	**29**	**1.2**	**0.0**	**0.0**	**0.0**	
**Terpene**	**30**	**0.0**	**0.0**	**4.5**	**0.0**	
**Terpene**	**31**	**0.0**	**0.0**	**4.5**	**0.0**	
**Terpene**	**32**	**0.0**	**0.0**	**4.5**	**0.0**	
**Terpene**	**33**	**0.0**	**0.0**	**4.5**	**0.0**	
**Terpene**	**34**	**0.0**	**0.0**	**4.5**	**0.0**	
**Terpene**	**35**	**0.0**	**0.0**	**4.5**	**0.0**	
**Terpene**	**36**	**0.0**	**0.0**	**4.5**	**0.0**	
Terpene	37	0.0	0.0	0.0	1.6	
Terpene	38	0.0	0.0	0.0	1.6	
Terpene	39	0.0	0.0	0.0	1.6	
Terpene	40	0.0	0.0	0.0	1.6	
Terpene	41	0.0	0.0	0.0	1.6	
Terpene	42	0.0	0.0	0.0	1.6	
Terpene	43	0.0	0.0	0.0	1.6	
Terpene	44	0.0	0.0	0.0	1.6	
Terpene	45	0.0	0.0	0.0	1.6	
Terpene	46	0.0	0.0	0.0	1.6	
Terpene	47	0.0	0.0	0.0	1.6	
Terpene	48	0.0	0.0	0.0	1.6	
Terpene	49	0.0	0.0	0.0	1.6	
Terpene	50	0.0	0.0	0.0	1.6	
Terpene	51	0.0	0.0	0.0	1.6	
Terpene	52	0.0	0.0	0.0	1.6	
Terpene	53	0.0	0.0	0.0	1.6	

Bolded rows indicate GCFs found in only *Alternaria* genomes.

SUPPLEMENTARY TEXT

Gene prediction parameter testing

Parameter optimization during this gene prediction step critically affects downstream analyses, since antiSMASH, BiG-SCAPE, and CytoScape build upon these predicted genes as inputs. Reliable gene predictions lead to reliable conclusions. For this reason, thorough testing and comparison of gene prediction parameters were performed, as outlined below. A summary of the gene counts recovered from each trial can be found in Table S3**.**

Alternaria genome processing

Gene prediction modules in funannotate were first trained to optimize their parameters for predicting genes in *Alternaria* genomes.

Trial 1

The goal of Trial 1 was to optimize the <funannotate predict> command so that gene prediction results for the *A. alternata* SRC1lrK2f genome similarly matched reported gene counts from NCBI database records of 13,466 genes (Accession: GCA_001642055.1). Multiple iterations of the command were tested on the *A. alternata* SRC1lrK2f genome with modifications made to the base command. The gene predictors called in <funannotate predict> are Augustus, snap, glimmerHMM, CodingQuarry and GeneMark-ES. EvidenceModeler creates a consensus gene model based on all the “evidence” presented from each program, taking into account their assigned weightings. The heavier the weight, the more the gene predictor influences the final consensus model. By default, all inputs are set to 1.

The baseline was established by first running <funannotate predict> *ab initio*, on its default settings without modification or training; 10,941 gene models were reported.

In the second iteration, Augustus was pre-trained using the <funannotate train> command. The remaining gene predictors only analyze genomic sequences and are not

trained in advance. Transcriptomic RNA-seq data from the same strain [A. alternata SRC1lrK2f; Accession: SRR4063372; 1, 2] undergo *de novo* genome-guided assembly via Trinity, which PASA uses to create RNA-seq generated gene models. The resulting gene models are used to create a set of pre-trained parameters that can be invoked by indicating the <--species> flag when using <funannotate predict>. Augustus then uses the species-specific parameters to predict gene models in the query genome. Going forward, prediction with pre-trained species parameters for *A. alternata* will be referred to as “trained gene prediction”. Additionally, the output, a “transcript evidence” file, can be provided to <funannotate predict> to better identifying gene, intron, and exon boundaries. Trained gene prediction with the default weightings was performed, and 11,089 gene models were reported.

Results from the second iteration showed that the gene prediction tool snap performed poorly (23 gene predictions) and negatively impacted overall gene prediction performance using funannotate. It is now known that the conda distribution of snap that was bundled along with funannotate ver. 1.8.7 was broken, so gene prediction evidence from snap was weighted at 0 such that it would not affect the reported overall gene counts. GlimmerHMM is also known to have relatively low specificity when performing gene prediction on nucleotide data [3]. However, when both snap and glimmerhmm were weighted at 0, funannotate failed due to an index error within Evidence Modeler. Moving forward, only snap was weighted at 0.

In the third attempt, trained gene prediction was re-done with snap weighted at 0 and without transcript evidence; 12,093 gene models were reported. When transcript evidence from the training step was provided, 12,234 gene models were predicted. Finally, trained gene prediction with CodingQuarry and PASA (high-quality gene models in gff3 format) reported 13,372 genes. Unable to provide further training or evidence and having only <100 gene difference from the reported gene counts, Trial 1 was considered satisfactory and concluded.

Trial 2

In Trial 2, the best parameters from Trial 1 were applied to a closely related genome from *Alternaria* section *Alternaria* to determine whether or not Trial 1 parameters were over-fit to *A. alternata*. *A. tenuissima* FERA1166 (Accession: GCA_004156035.1) was selected for this trial. NCBI reported 13,566 predicted genes in the assembly.

Ab initio gene prediction on default parameters reported 11,534 genes. Meanwhile, the second-best parameters from Trial 1 (trained gene prediction with transcript evidence and snap weighted at 0) resulted in 12,729 gene models - a 837 gene difference. Training on *A. alternata* SRC1lrK2f improves gene prediction performance in closely related species.

To mimic the best parameters from Trial 1, CodingQuarry and PASA were activated. However, gene prediction in genomes that were not the original genome (*A. alternata* SRC1lrK2f) failed because the FASTA headers in the RNA BAM file did not match the FASTA headers in the newly provided genome file. For Trial 2, pre-trained parameters with <--transcript_evidence> were deemed best for prediction.

Trial 3

Trial 3 was performed to test another closely related *Alternaria* species, *A. gaisen* FERA650, for the same purpose as described for Trial 2 above. *A. gaisen* is also in *Alternaria* section *Alternaria* but more distantly related to the training species than the *A. tenuissima*. NCBI reported 13,150 genes in the *A. gaisen* strain FERA650 assembly (Accession: GCA_004156025.2).

Ab initio gene prediction on default parameters reported 11,220 gene models, and the best parameters from Trial 2 (with transcript evidence provided and snap weighted at 0) resulted in 12,386 gene models. It is demonstrated again that gene prediction in *Alternaria* returns the better results when given transcript evidence, pre-trained parameters, and snap: 0, although there is still a 764 gene difference with the reported counts in NCBI.

The predicted gene counts for Trials 2 and 3 on the “best” pre-trained parameters are much lower than their reported counts, compared to the results from Trial 1. This can be explained by transcriptomic and genomic differences between species. The trained parameters are tuned to the particular characteristics of *A. alternata* SRC1lrK2f. The parameters are expected to perform well for the same species with the same characteristics and with less accuracy as they are applied to more distantly related species, as with *A. tenuissima* and *A. gaisen*. Differences in the number of reported and predicted genes could also be due to differences in defaults, biases, and/or random elements inherent to different gene prediction programs, versions, and pipelines. For these reasons, the *A. alternata* pre-trained parameters with transcript evidence were considered satisfactory and used for gene prediction on *Alternaria* genomes.

Non-Alternaria (other Pleosporaceae) genome processing

Trial 4

It was evident that genus-specific parameter pre-training improved gene prediction performance in the three *Alternaria* species tested in Trials 1-3*.* The next step was to test how gene prediction performance in non-*Alternaria* assemblies would change with (1) *ab initio* prediction, (2) pre-trained parameters from *A. alternata* SRC1lrK2f transcriptomic data, and (3) pre-trained parameters from genus-specific transcriptomic data. The *Decorospora gaudefroyi* P77 genome (Accession: GCA_004156025.2; 11,835 genes) and transcriptome (Accession: SRX2770397) were used as the test specimen.

Ab initio gene prediction with snap weighted at 0 reported 10,294 genes. Gene prediction trained on *A. alternata* strain SRC1lrK2f with transcript evidence and snap weighted at 0 resulted in 10,246 genes. Gene prediction with parameters trained on the *D. gaudefroyi* strain P77 transcriptome with transcript evidence provided and snap weighted at 0 resulted in 10,631 genes, which was still 1,204 genes fewer than the reported count.

In this trial, gene predictor parameters trained on *A. alternata* did not improve compared to *ab initio* gene prediction in *D. gaudefroyi*. Although the number of predicted genes increased by 385 after training on the *Decorospora* transcriptome, the process was time-consuming and evidently did not always perform as well as expected. Therefore, genus-specific training was not performed for non-*Alternaria* genomes, which were also not the priority of this study. Moving forward, *ab initio* gene prediction was done for non-*Alternaria* genomes with evidence from snap weighted at 0.

Curation of genome sample and taxon names

During the processing of genomes, funannotate detected several "bad contigs" (i.e., contigs that did not contain all four nucleotides) in the genomes of *A. alternata* ATCC34957 (Accession: GCA_001443195.1), *A. chartarum* KAS5825 (Accession: KAS5825), and *Pyrenophora tritici* 86-124 (Accession: GCA_003231425.1). It is possible these contigs, which were typically quite short, contained repetitive regions in the genome involving only three nucleotides. In each case, the bad contig was manually erased, the genomes were saved under a new accession number (GCA_001443195.2, GCA_003231425.2, and KAS5825-2, respectively), and processed again with funannotate.

Inference of the large-scale phylogeny was performed on whole-genome data using Orthofinder and 1,101 shared, single-copy orthogroups. Strains that displayed inconsistent behaviour were flagged for further investigation. Three strains (*Curvularia kusanoi* 30M1, *Bipolaris sorokiniana* WAI2411, and *B. sorokiniana* WAI2406) had very long branch lengths and did not cluster with other members of their genus as expected. Further analyses of the housekeeping gene *rpb2* from *C. kusanoi* 30M1 (data now shown) indicated the strain likely belongs to the genus *Epicoccum* in the *Didymellaceae*. These three assemblies with dubious taxonomic identities were excluded from our final genome sample.

In other cases, the inconsistency could be reconciled by synonymy of different names previously given to the sexual and asexual states of the same fungus. For instance,

Cochliolobolus and *Bipolaris* can often refer to the same fungus [4], but modern taxonomy has shifted to eliminate this redundancy. Some updates of species names were also made based on recent taxonomic revisions or previous analyses of particular strains. Below we list the nomenclatural changes made to original names that were given at the time of genome accessioning:

Alternaria tenuissima → *Alternaria alternata*
Alternaria citriarbusti → *Alternaria alternata*
Alternaria limoniasperae → *Alternaria alternata*
Alternaria mali → *Alternaria alternata*
Alternaria fragariae strain BMP3062 → *Alternaria gaisen strain* BMP3062
Alternaria mali strain BMP3063 → *Alternaria arborescens strain* BMP3063
Alternaria alternata EV-MIL-31 → *Alternaria longipes strain* EV-MIL-31
Alternaria tangelonis strain BMP2327 → *Alternaria longipes strain* BMP2327
Cochliobolus heterostrophus strain C4-1 → *Bipolaris maydis strain* C4-1
Cochliobolus heterostrophus strain Hm540-1 → *Bipolaris maydis strain* Hm540-1
Cochliobolus lunatus strain m118 → *Curvularia lunata strain* m118
Exserohilum rostratum strain ER1 → *Setosphaeria rostrata strain* ER1
Exserohilum turcicum strain NY001 → *Setosphaeria turcica strain* NY001

SUPPLEMENTARY REFERENCES

1. Santelli CM, Pfister DH, Lazarus D, Sun L, Burgos WD, Hansel CM. Promotion of Mn(II) oxidation and remediation of coal mine drainage in passive treatment systems by diverse fungal and bacterial communities. Appl Environ Microbiol. 2010;76(14):4871-5.

2. Zeiner CA, Purvine SO, Zink EM, Paša-Tolić L, Chaput DL, Haridas S, et al. Comparative Analysis of Secretome Profiles of Manganese(II)-Oxidizing Ascomycete Fungi. PLoS One. 2016;11(7):e0157844.

3. Scalzitti N, Jeannin-Girardon A, Collet P, Poch O, Thompson JD. A benchmark study of ab initio gene prediction methods in diverse eukaryotic organisms. BMC Genomics. 2020;21(1):293.

4. Manamgoda DS, Cai L, McKenzie EHC, Crous PW, Madrid H, Chukeatirote E, et al. A phylogenetic and taxonomic re-evaluation of the Bipolaris - Cochliobolus - Curvularia Complex. Fungal Divers. 2012;56(1):131-44.
